# Bottom trawl catch comparison in the Mediterranean Sea: Flexible Turtle Excluder Device (TED) *vs* traditional gear

**DOI:** 10.1371/journal.pone.0216023

**Published:** 2019-12-04

**Authors:** Claudio Vasapollo, Massimo Virgili, Andrea Petetta, Giada Bargione, Antonello Sala, Alessandro Lucchetti

**Affiliations:** 1 National Research Council (CNR), Institute of Biological Resources and Marine Biotechnologies (IRBIM), Largo Fiera della Pesca, Ancona, Italy; 2 Department of Biological, Geological and Environmental Sciences, University of Bologna, Piazza di Porta San Donato, Bologna, Italy; Universidad de Cádiz, Facultad de Ciencias del Mar y Ambientales, SPAIN

## Abstract

The Mediterranean Sea is a biodiversity hotspot where intense fishing pressure is associated with high bycatch rates of protected species (sea turtles and cetaceans) and top predators (sharks). Since the conservation of these species has become a priority, fishery scientists are faced with the challenge of reducing incidental catch, which entails high rates of mortality. Among the species threatened by fishing activities, the loggerhead turtle (*Caretta caretta*) is a charismatic species considered as “vulnerable” at the global scale. In the Mediterranean Sea trawl nets are the gears with the highest probability of catching protected species incidentally. A new flexible Turtle Excluder Device (TED) was tested for the first time on commercial bottom trawlers to assess its effectiveness in reducing bycatch in the Mediterranean Sea. Analysis of the total catches of the hauls made with and without the TED showed that the difference in terms of weight was not significant. The catch of the main commercial species showed similar rates without a significant loss of size (i.e. total length) with the exception of the largest anglerfish (*Lophius* spp.). The bycatch of control nets included mostly rays and sharks, but never turtles, although the authors learned from the crews of other vessels operating in the same areas at the time of the trials that they had caught some loggerhead turtles. Our study demonstrates that TED scan be adopted without significantly affecting commercial catch. This informs fishers and managers for a practical and effective means that may reduce the bycatch of threatened species in coastal Mediterranean demersal multispecies fisheries. The measures involving gear modifications require significant investment but they are technically feasible and are capable of improving the conservation prospects of these endangered species. Besides ensuring normal earnings, the TED induced a significant reduction of debris and litter in the codend, thus reducing catch sorting time and improving catch quality.

## Introduction

The Mediterranean basin is considered as a biodiversity hotspot [1]. The Mediterranean fishing fleet is highly diversified and targets a wide range of species, but the intense fishing effort has resulted in resource overexploitation [2], deterioration of marine ecosystem services (e.g., in terms of goods and resources, such as food for millions of people) [3,4], and incidental catch of protected species, such as cetaceans [5], sea turtles [6] and top predators like sharks [7]. Bycatch reduction has become a key objective for fishery scientists as species conservation has become a priority for major international organizations. Among other measures, the Habitats Directive [8] has introduced a conservation policy aimed at reducing the bycatch of the species listed in Annex IV. The FAO International Guidelines on Bycatch Management and Reduction of Discards [9] envisage management measures for the conservation of target as well as non-target species. However, conservation aims are often hampered by competing social, economic, ecological factors as well as by fishery management objectives that do not acknowledge the migratory nature of the main endangered species [10].

Among the animals threatened by fishing activities, the loggerhead sea turtle (*Caretta caretta*) is a charismatic species considered as “vulnerable” at the global scale [11]. While considered “least concern” in the Mediterranean Sea [12], the adoption of effective actions for loggerhead turtle conservation in the Mediterranean is essential given their life stages and migrations. This vulnerable species’ movements to and from breeding and feeding grounds place them at high risk of interaction with several types of fishing gears, including towed gears, set nets, and longlines [13,14]. According to Casale [15] more than 150,000 loggerheads are caught incidentally each year in the Mediterranean, with a mortality of more than 50,000. Lucchetti et al. [13] have recently estimated that more than 52,000 turtles are caught yearly along the Italian coasts and that about 10,000 die.

Trawl nets are the gears involving the highest bycatch probability, also of loggerhead turtles [16]. They raise special concern in the Adriatic Sea, whose shallow waters are favourable fishing grounds exploited by more than 1,000 bottom trawlers, mainly from Italian and Croatian commercial fleets [14] and foraging areas rich in benthic communities for sea turtles [17]. The massive presence of both fishing vessels and loggerheads makes the north-western Adriatic a bycatch hotspot, especially in late summer and autumn [16]. The annual trawler bycatch of sea turtles in the northern Adriatic Sea has been estimated to exceed 6,500 individuals [13,18].

In the late 1980s, a technical measure to reduce sea turtle bycatch began to be introduced in U.S. fisheries [19,20]. The Turtle Excluder Device (TED) is a grid that stops large objects or animals from entering the codend and allows them to swim out of the net through an opening set before it. Because of their effectiveness, which has mainly been demonstrated in prawn trawl fisheries, TEDs have become mandatory in several countries [14]. In the view of Casale et al. [18], TEDs would prove less efficient in the Mediterranean, because they would also exclude the larger individuals of several commercial species (e.g., angler fishes and cods). Yet, recent experiments conducted in the Mediterranean Sea did not show any loss in terms of commercial catches [21–24]. Moreover, the TED reduced debris in the codend, improving catch quality and shortening onboard sorting operations, thus increasing fishing time and earnings [21].

Based on these considerations, a study was conducted to test the effectiveness of a TED by fitting it in the nets of commercial trawlers targeting different species of the northern Adriatic Sea. Its aims were: 1) to study the general gear performance with and without the TED, whatever the season and fishing conditions; 2) to compare the catch rates of commercial species, discards and debris (either anthropogenic and natural;, and 3) to analyse any TED-related size selection by length-based analysis of the main commercial species.

## Materials and methods

### Sea trials

**Fig 1:**
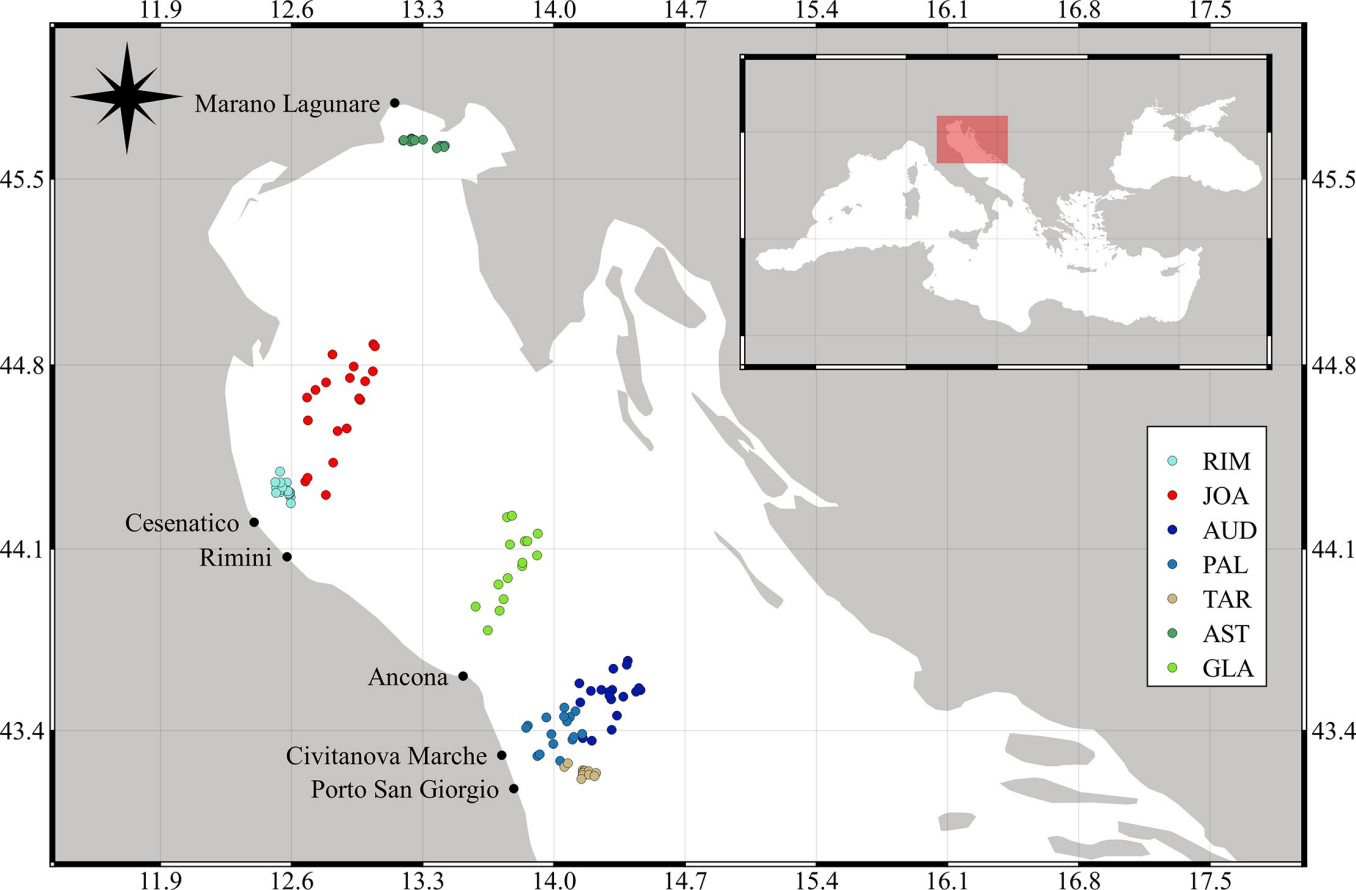
Study area, hauls made during the sea trials, and base harbour of each vessel. AUD: Audace, RIM: Rimas, JOA: Joacchì, AST: Astuzia, GLA: Gladiatore, PAL: Palestini, TAR: Tarantini.

Seven bottom trawlers from different Italian harbours in the northern and central Adriatic Sea were randomly selected for the sea trials (Fig 1). Five vessels performed paired cruises, one with and one without the TED. Each trial, with and without TED, was performed in the same area and very close temporally (in a couple of weeks, maximum) to reduce temporal biases and keep experimental conditions as similar as possible. The other 2 vessels were twin trawlers, where one net was equipped with the TED and the other (control, CTRL) was not. The vessels were coded based on the first three letters of their name as follows: AUD = Audace (twin trawler), RIM = Rimas, JOA = Joachì, AST = Astuzia, GLA = Gladiatore (twin trawler), PAL = Palestini, and TAR = Tarantini. The trials, which were conducted in 2015, 2016 and 2017 from June to December (Table 1), were part of their routine fishing activities. The scientific observers made measurements onboard.

**Table 1:** Main characteristics of the trawlers used in the sea trials. AUD: Audace, RIM: Rimas, JOA: Joacchì, AST: Astuzia, GLA: Gladiatore, PAL: Palestini, TAR: Tarantini. LOA: Length overall, GT: Gross Tonnage.

Vessel	Power [kwh]	LOA [m]	GT [ton]	Month	Year
AUD	872	26.8	130	July	2015
RIM	142	13.6	15	November	2015
JOA	574	26.2	96	March	2016
AST	147	15.5	16	June	2016
GLA	870	26.5	96	December	2016
PAL	206	22.3	50	April	2017
TAR	167.9	17.3	24	July	2017

### TED specifications

**Fig 2:**
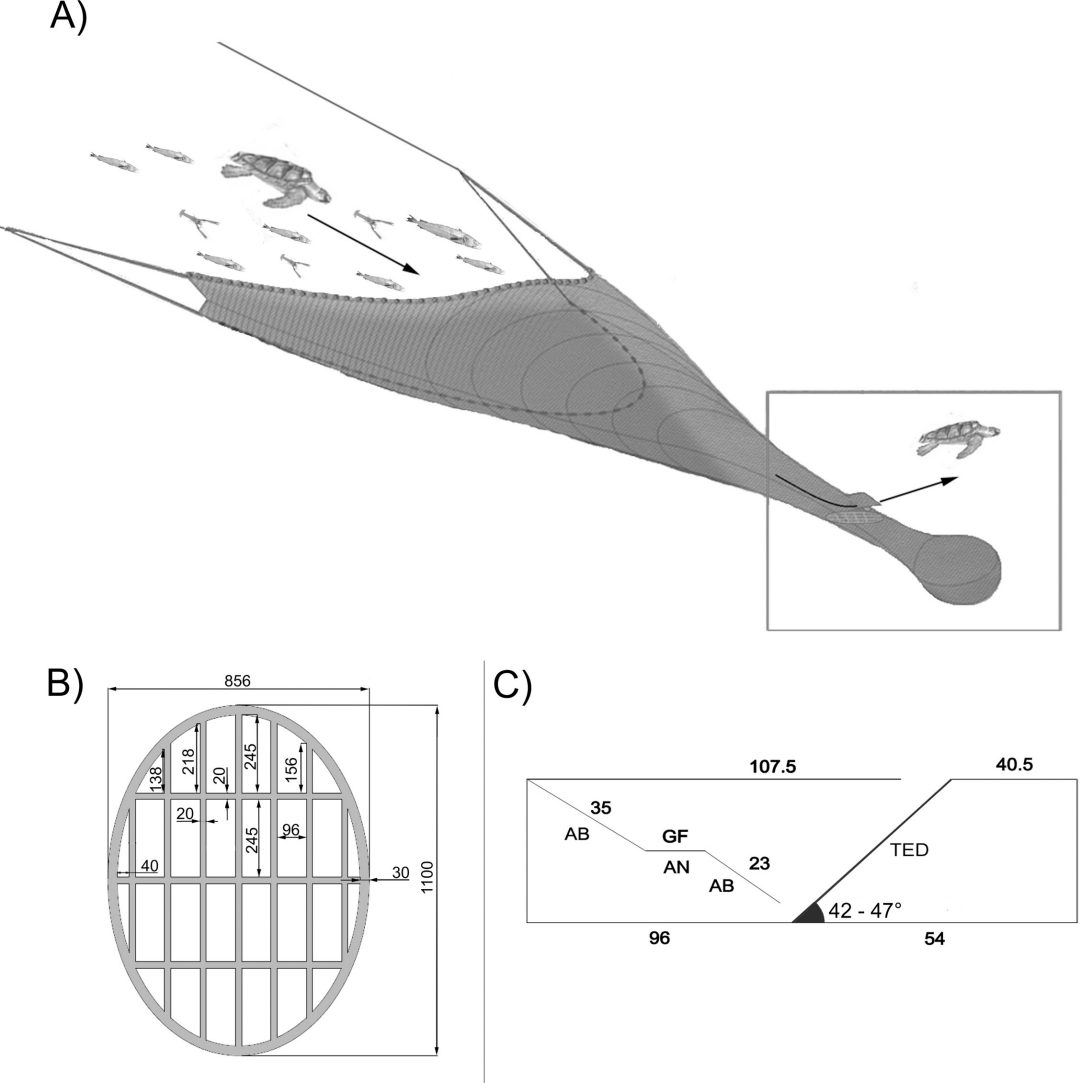
The flexible Turtle Excluder Device (TED). a) Position of the TED in relation to the codend. b) Specification of the high-strength, flexible plastic TED used in the trials. Measurements given in mm. c) Technical drawing of the TED rigging (lateral view). GF: Guide Funnel. AB and AN, types of net cuts (AB is a parallel cut to a line of sequential mesh bars; AN is a cut perpendicular to the general course of the net; figures indicate mesh numbers). The mean grid angle recorded during sampling is also reported.

The flexible TED used for these trials was made of high-strength plastic material and was designed in house according to the technical specifications suggested by Mitchell et al. [25] (Fig 2). It was mounted on a tubular netting section (6 m in length) with a tilt angle of approximately 46° and placed in the extension piece, just in front of the codend. An opening was cut into the upper portion of the net, just before the TED, and covered with a netting panel of which three sides were sewn to the net, to prevent the escape of commercial species. The panel works like a valve, opening when it is hit by large and heavy objects, and allows sea turtles and other bycatch species to swim out of the net. In line with the specifications suggested by Mitchell et al. [25], an accelerator funnel was installed before the TED to drive the fish down and away from the exit, through the TED and towards the codend.

The TED angle is a key factor influencing TED efficiency and preventing the loss of commercial species during towing [25,26]. An angle less than 40° may involve catch loss due to water diversion through the exit hole, whereas angles greater than 55° can prevent turtle escape and deflection of trash, thus clogging the grid. The TED angle was sampled at 60 s intervals by Star Oddi Data Storage Tags (DST) sensors (Iceland) mounted directly on the grid.

TED performance, fish reactions to the grid and fish behaviour in the net were monitored with a GoPro Hero4 underwater camera. Due to the high water turbidity, the camera was mounted 1 m from the TED. The fisheye lens constantly provided a full view of the TED, monitoring its position during towing.

### Catch analysis

The catch of each haul was subdivided into commercially important species, discards (invertebrate and fish species of no commercial value and specimens under the legal size), and debris (anthropogenic litter and natural material like stones and wood).

Catches were standardized based on the formulae:

$${CPUE}_{W}=W/\left(60\prime /Trawl\;Duration\right)$$ (1)

$${CPUE}_{I}=Ind/\left(60\prime /Trawl\;Duration\right)$$ (2)

where *CPUE*_*W*_ is the catch per unit effort expressed as weight (kg) per hour of trawling, *CPUE*_*I*_ is the catch expressed in terms of individuals caught per hour, *W* is the weight of the catch of each haul, and *trawl duration* is net fishing time in minutes. Wilcoxon’s signed rank test was used to assess differences in mean catches obtained by each vessel with/without the TED, trawl duration and fishing depth [27].

A Generalized Linear Mixed Model (GLMM) was employed to compare commercial CPUE_W_, discards and debris. Three independent variables, net (“TED” *vs* “CTRL”), depth (“low”, 11–30 m; “medium”, 30–50 m; “high”, 50–90 m), and year were first tested for co-linearity either visually (with a scatterplot of each variable *vs* each of the others) and by Pearson’s correlations. The vessel term was considered as a random factor. Model selection was based on Akaike’s Information Criterion (AIC) and the Log Likelihood Ratio Test, according to the protocol in Zuur et al. [28]. The trends of residuals and heteroscedasticity were assessed to check whether statistical assumptions were respected. If variance heterogeneity was associated with a variable, the variance structure of the model was modified to take into account a different variance for each level of the variable [28,29]. When one or more factors of the models were significant (p < 0.05), a pairwise test based on Tukey’s test was applied to identify the levels showing significantly different mean values.

For the commercial species, the total length (TL) of each specimen was measured onboard, rounded down to the nearest 0.5 cm. To assess the TED’s influence on the size of the fish caught, the length frequency distributions (LFDs) of the commercial species accounting for more than 5% of the total catch in weight were analysed for each vessel. The catch comparison to assess the catch efficiency of the TED relative to the CTRL nets was made using the GLMM. The probability of a fish being retained by the TED follows from:

$$Pr\{TED/(TED+CTRL)\}=1/\left(1+e^{-(\beta_{0}+\beta_{1}\times length+\beta_{2}\times {length}^{2}+\beta_{3}\times {length}^{3})}\right)$$ (3)

A binomial error distribution was used to calculate the probability of the number of fish caught in the TED net based on 1-cm size classes. A probability value of 0.5 corresponds to equal catches in both nets. According to Holst and Revill [30], a 3^rd^ order polynomial would be adequate in most cases, although in some instances a 1^st^ or 2^nd^ order would be sufficient. The best binomial model was chosen based on the AIC. In models like this, usually a random term is added to the models (generally the hauls) [30–34]. In our study, since there was no obvious way of pairing the data from the individual TED and CTRL hauls (some of the vessels had different, although negligible, number of hauls; Table 2), the hauls of each trawler were pooled together [35] and the term vessel was used as a random intercept. Moreover, as the TL of the individuals of some species was not consistent across vessels, TL was used as a random slope. Since the catch was related to the time of the year, the amount of the target species in the catch varied. As a consequence, the model for each species was run with a different number of vessels. The models are reported with a 95% confidence interval calculated by the bootstrap method with 999 simulations.

**Table 2:** Operating conditions and catch per unit effort based on weight (kg h^-1^) for each vessel during the sea trials. SE: standard error, ns: not significant. P value: Wilcoxon test. AUD: Audace, RIM: Rimas, JOA: Joacchì, AST: Astuzia, GLA: Gladiatore, PAL: Palestini, TAR: Tarantini.

			Tilt angle [degree]		Tow duration [min]				Depth [m]				Commercial catch [kg h^-1^]				Discard catch [kg h^-1^]				Debris catch [kg h^-1^]			
Vessel	Net Type	No. Hauls	MEAN	SE	MEAN	SE	Range	p value	MEAN	SE	Range	p value	MEAN	SE	Range	p value	MEAN	SE	Range	p value	MEAN	SE	Range	p value
AUD	TED	18	46.0	0.7	139.2	1.0	97–169	ns	78.9	0.3	67.3–88.0	ns	10.2	0.7	5.4–15.3	ns	9.6	0.9	4.4–18.4	<< 0.001	5.3	0.6	1.7–10.8	<< 0.001
	CTRL	18			139.2	1.0	97–169		78.9	0.3	67.3–88.0		13.3	1.2	7.4–28.7		12.8	1.4	3.7–24.1		10.6	1.1	4.2–21.1	
RIM	TED	10	44.9	0.9	139.4	1.2	122–166	ns	18.7	0.3	12.1–23.7	ns	40.0	2.3	28.4–50.7	ns	17.7	1.5	12.3–26.7	ns	3.8	0.6	1.7–7.3	ns
	CTRL	10			143.2	1.3	113–167		18.2	0.2	13.0–21.6		40.3	3.2	24.6–56.4		15.5	2.0	7.8–29.8		5.7	1.1	1.3–10.1	
JOA	TED	10	41.5	0.5	116.0	0.7	100–130	ns	34.1	0.4	20.0–38.3	0.033	13.7	1.1	8.8–19.9	ns	12.2	2.2	36.8–41.2	ns	4.5	1.1	0.7–9.9	ns
	CTRL	9			109.1	0.7	95–130		30.6	0.2	25.0–35.0		14.6	0.7	10.7–17.4		11.7	20.6	4.4–30.4		4.0	1.3	0.5–12.1	
AST	TED	10	42.4	0.6	66.1	1.3	50–100	ns	13.5	0.1	12.9–15.4	ns	12.2	1.9	4.7–24.9	ns	126.8	27.6	18.6–271.5	ns	36.6	9.9	5.4–109.7	0.048
	CTRL	8			84.2	2.5	45–115		12.6	0.1	11.9–15.0		11.7	3.5	4.4–30.4		107.0	44.2	36.9–348.7		21.5	10.8	4.4–84.5	
GLA	TED	15	47.1	0.7	117.3	0.5	95–132	ns	39.1	0.2	26.0–43.2	ns	21.6	1.4	12.5–32.5	ns	22.7	3.7	7.7–47.8	0.046	1.5	0.3	0.0–3.3	0.002
	CTRL	15			117.3	0.5	95–133		39.1	0.2	26.0–43.2		25.0	1.3	16.2–35.0		31.3	4.6	7.8–59.1		5.2	1.0	1.6–17.3	
PAL	TED	8	43.5	0.9	145.8	1.8	130–200	ns	49.3	0.9	21.4–61.0	ns	10.5	1.0	6.5–14.0	ns	5.5	0.8	3.1–8.9	0.018	8.5	0.9	3.9–12.7	ns
	CTRL	8			133.9	0.6	130–150		46.3	1.3	23.0–66.0		7.4	0.8	4.5–11.1		8.6	1.6	10.7–15.6		10.0	1.8	1.8–16.6	
TAR	TED	7	44.6	1.5	146.6	0.4	135–155	0.048	62.1	1.1	30.0–73.5	ns	12.4	0.8	10.1–15.9	ns	23.2	0.8	19.2–26.3	ns	9.7	1.4	4.6–13.9	ns
	CTRL	7			140.2	0.3	135–146		61.9	1.1	34.6–74.3		13.1	0.6	10.7–15.6		17.3	2.2	12.3–126.5		15.9	2.6	9.5–29.7	

All analyses were performed with the free software R [36] and the R packages *nlme* [37] and *lme4* [38].

### Ethic statements

All observer embarkations on the fishing vessels have been authorised by the coastguard of the respective ports. Moreover, the field study did not involve endangered or protected species.

## Results

The efficiency of the TED *vs* the CTRL nets was assessed based on data from 153 hauls (Table 2). According to the Wilcoxon test there were no differences in mean tow duration between TED and CTRL trawls, except that TAR showed a barely significant difference of about 6 minutes (Table 2). The mean fishing depth did not show significant differences with one exception, JOA, although this result may have been affected by the unequal number of TED and CTRL hauls.

### Gear performance

The underwater camera images and the sensor data showed that the TED did not affect net functioning in any vessel. The TED tilt angle (Table 2), obtained from > 7,200 pings (> 120 hours) per vessel, ranged from a mean value (± standard error, hereafter) of 41.5° ± 0.5° to 47.1° ± 0.7°.

### Catch rates

**Fig 3:**
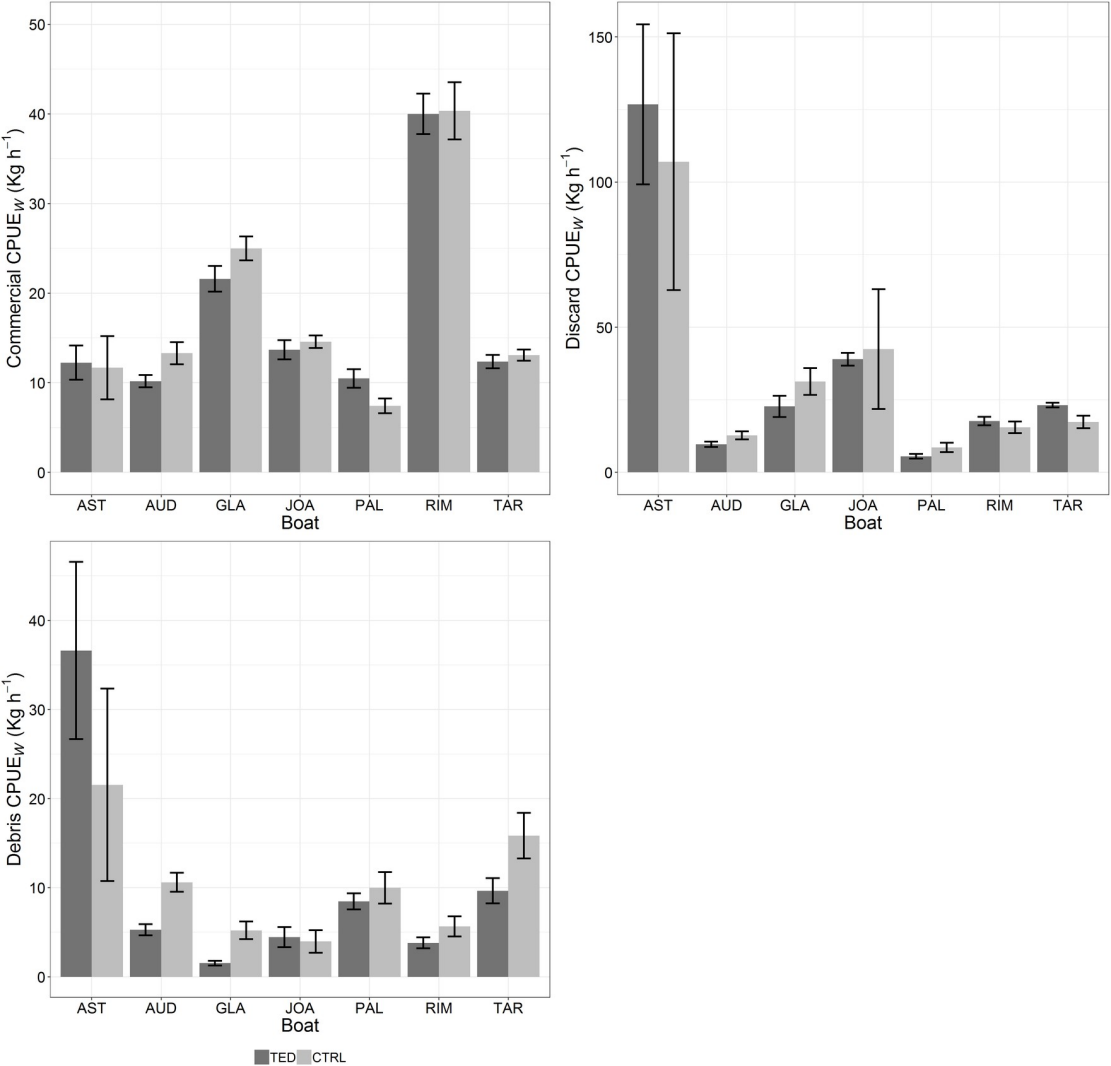
Mean commercial, discard and debris CPUE_W_ of TED and CTRL nets per vessel. Bars: standard error. AUD: Audace, RIM: Rimas, JOA: Joacchì, AST: Astuzia, GLA: Gladiatore, PAL: Palestini, TAR: Tarantini.

The catch rates are summarised in Table 2. The Wilcoxson test did not highlight any differences between the TED and the CTRL net in the standardized commercial catch of any vessel. In contrast, differences between TED and CTRL were found in the weight of discards for AUD and PAL with values always lower in the TED than in the CTRL net. Conversely, the differences between TED and CTRL weight of discard for GLA was merely p = 0.05. Significant differences between TED and CTRL were also found for debris weight in two vessels, AUD and GLA, again with lower values in the TED nets; AST appeared to have caught more debris with the TED than the CTRL net, but the difference was merely p = 0.05. The data of the commercial species, discards and debris are listed in “S1–S3 Tables”, respectively. The CPUE_W_ means (± standard error) for commercial, discard and debris for each vessel are represented graphically in Fig 3.

The model selections to test the effects of the explanatory variables on CPUE_W_ of commercial, discard and debris catches are reported in Table 3. The best model for the overall total commercial catches included only net and the factor was not significant, indicating that CPUE_W_ were similar in the TED and the CTRL nets (Table 4). The best model for discards (Table 3) comprised only depth and year. According to the pairwise test based on the factor year the discard biomass was highest in 2016, although the difference was barely significant (Table 4). The pairwise test based on the factor depth showed that discards were significantly more abundant at low and medium fishing depth than at high depth (Table 4). The best model for debris comprised net, depth, and their interactions (Table 3). According to the pairwise test, the CPUE_W_ of the TED nets was significantly lower than the CPUE_W_ of the CTRL nets (Table 4). Significant differences were also found between medium and high fishing depth (Table 4). The pairwise test for the interaction term highlighted significant differences between TED and CTRL at medium fishing depth and at high depth (Table 4).

**Table 3:** Model selection for the catch rates. In bold between square brackets: the best model explained by the independent variables. The estimates of the factors obtained by the regression are also reported with the respective standard errors, degree of freedom and t values.

	Models	Equation	AIC	Excluded Term	L. ratio	df	p
Commercial	Full Model	[CPUEW ~ net + depth + year + net x depth + net x year]	997.2				
	M1	[CPUEW ~ net + depth + year + net x year]	992.5	net x depth	1.35	2	0.508
	M2	[CPUEW ~ net + year + net x year]	987.5	depth	1.00	2	0.607
	M3	[CPUEW ~ net + year]	983.8	net x year	2.23	2	0.328
	M4	[CPUEW ~ net]	980.5	year	2.74	2	0.254
	Null Model	[CPUEW ~ 1]	979.6	net	2.12	1	0.146
			Model estimates				
			Value	SE	DF	df	p
		Intercept	16.97	4.15	144	4.09	0.0001
	netCTRL	1.26	0.86	144	1.45	0.148
Discard	Full Model	[CPUE_W_ ~ net + depth + year + net x depth + net x year]	1199.1				
	M1	[CPUE_W_ ~ net + depth + year + net x year]	1195.1	net x depth	1.96	2	0.375
	M2	[CPUE_W_ ~ net + depth + year]	1191.5	net x year	2.41	2	0.300
	**M3**	**[CPUE**_**W**_ **~ depth + year]**	1188.5	net	0.03	1	0.0868
	M4	[CPUE_W_ ~ depth]	1189.2	year	6.71	2	0.035
	M5	[CPUE_W_ ~ year]	1190.3	depth	7.82	2	0.020
	Variance Structure = VarIdent(~1|depth)+VarIdent(~1|year)		Model estimates				
			Value	SE	df	t-value	p
		Intercept	10.97	7.59	135	1.44	0.151
		depthMedium	3.64	1.42	135	2.56	0.0116
		depthLow	5.86	2.44	135	2.41	0.0175
		year2016	35.10	12.10	4	2.90	0.044
	year2017	1.26	10.64	4	0.12	0.911
Debris	Full Model	[CPUE_W_ ~ net + depth + year + net x depth + net x year]	977.8				
	M1	[CPUE_W_ ~ net + depth + year + net x depth]	971.9	net x year	0.18	2	0.913
	**M2**	**[CPUE**_**W**_ **~ net + depth + net x depth]**	970.9	year	4.94	2	0.085
	M3	[CPUE_W_ ~ net + depth]	972.1	net x depth	7.26	2	0.027
	M4	[CPUE_W_ ~ net]	974.7	depth	8.58	2	0.014
	M5	[CPUE_W_ ~ depth]	998.8	net	29.6	1	<0.0001
	Variance Structure = VarIdent(~1|depth)+VarIdent(~1|year)						
			Model estimates				
			Value	SE	df	t-value	p
		Intercept	7.53	2.30	140	3.27	0.0013
		netCTRL	1.49	1.60	140	0.93	0.355
		depthMedium	-2.96	2.35	140	-1.26	0.2099
		depthHigh	-0.05	2.60	140	-0.02	0.9846
		netCTRL X depthMedium	0.88	1.71	140	0.51	0.609
		netCTRL X depthHigh	3.87	1.90	140	2.03	0.0441

**Table 4:** Raw means and standard errors (SE) for commercial, discard and debris catches. The significant results (except for factor “net” of the commercial catch) of the Tukey’s pairwise of the selected models are also reported.

	Factor	Levels	Mean	SE	Pairwise	
Commercial	net	CTRL	18.7	1.4	TED = CTRL	p = 0.148
		TED	17.1	1.2		
Discard	year	2016	56.7	0.8	2016 > 2015	p = 0.044
		2015	13.1	9.3	2016 > 2017	p = 0.049
		2017	13.2	1.5		
	depth	High	12.1	0.9	Low > High	p = 0.018
		Medium	30.2	5.4	Medium > High	p = 0.012
		Low	51.4	10.3		
Debris	net	TED	9.0	1.8	TED = CTRL	p < 0.001
		CTRL	9.5	1.2		
	depth	Low	13.3	2.9	Medium < High	
		Medium	4.2	0.5		p = 0.006
		High	9.5	0.7		
	net X depth	TED X Low	17.0	5.1	TED X Medium < CTRL X Medium	
		CTRL X Low	9.8	3.1		p = 0.001
		TED X Medium	3.2	0.6		
		CTRL X Medium	5.5	0.8	TED X High < CTRL X High	
		TED X High	6.9	0.7		p < 0.001
		CTRL X High	12.2	1.1		

**Fig 4:**
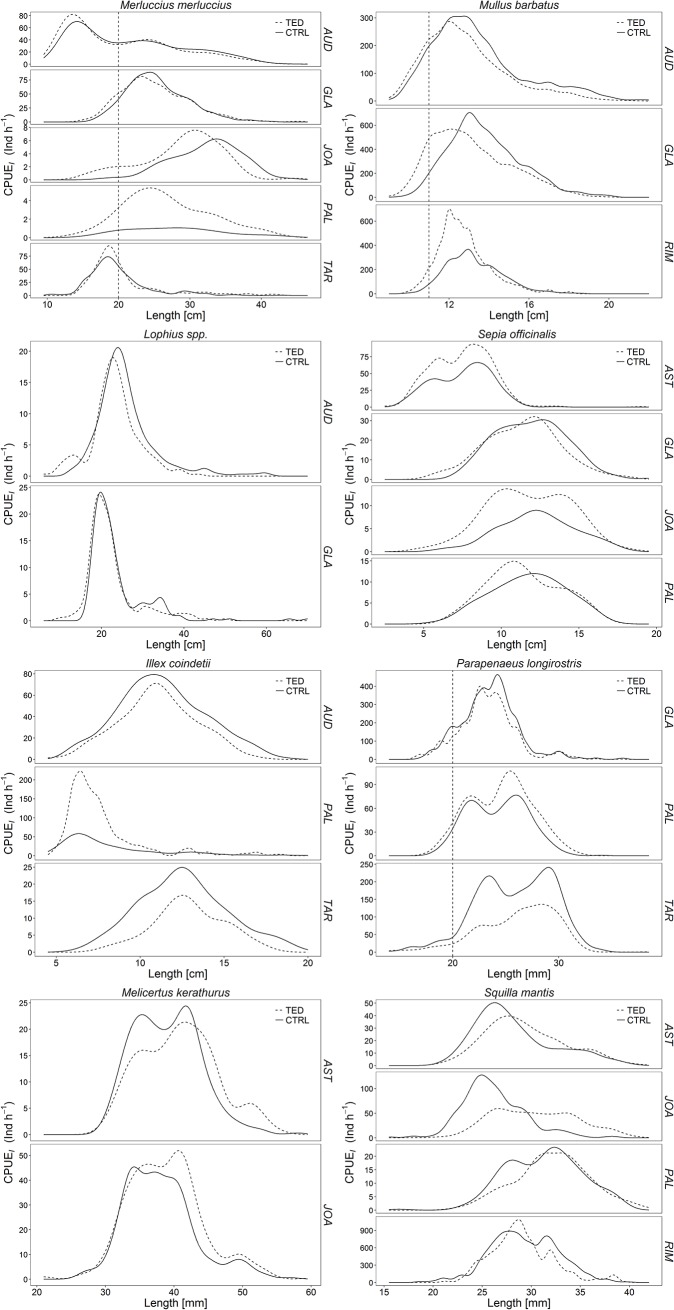
Catch length-frequency distributions of each commercially important species per vessel. AUD: Audace, RIM: Rimas, JOA: Joacchì, AST: Astuzia, GLA: Gladiatore, PAL: Palestini, TAR: Tarantini.

**Fig 5:**
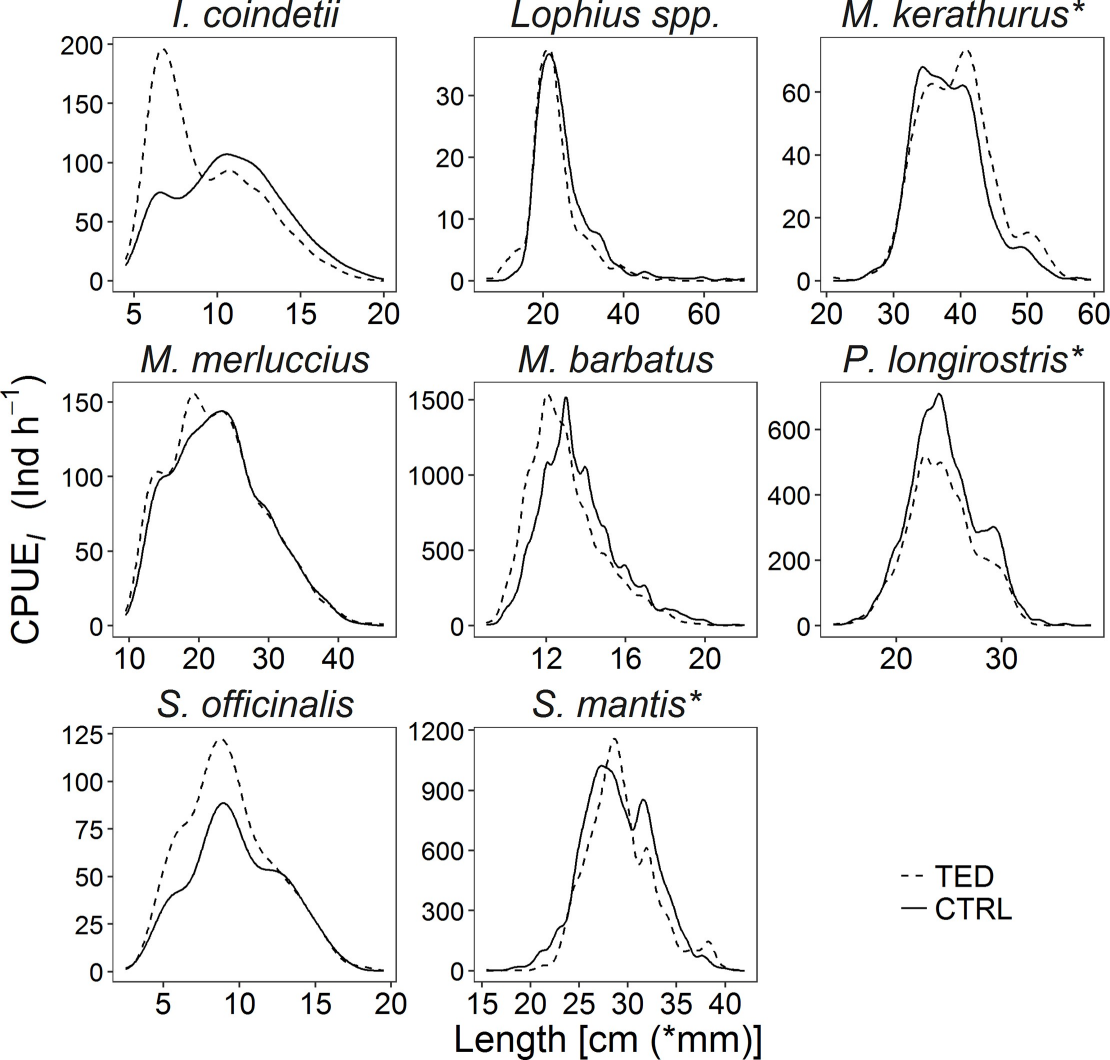
Pooled catch length-frequency distribution of each commercially important species (all vessels).

**Fig 6:**
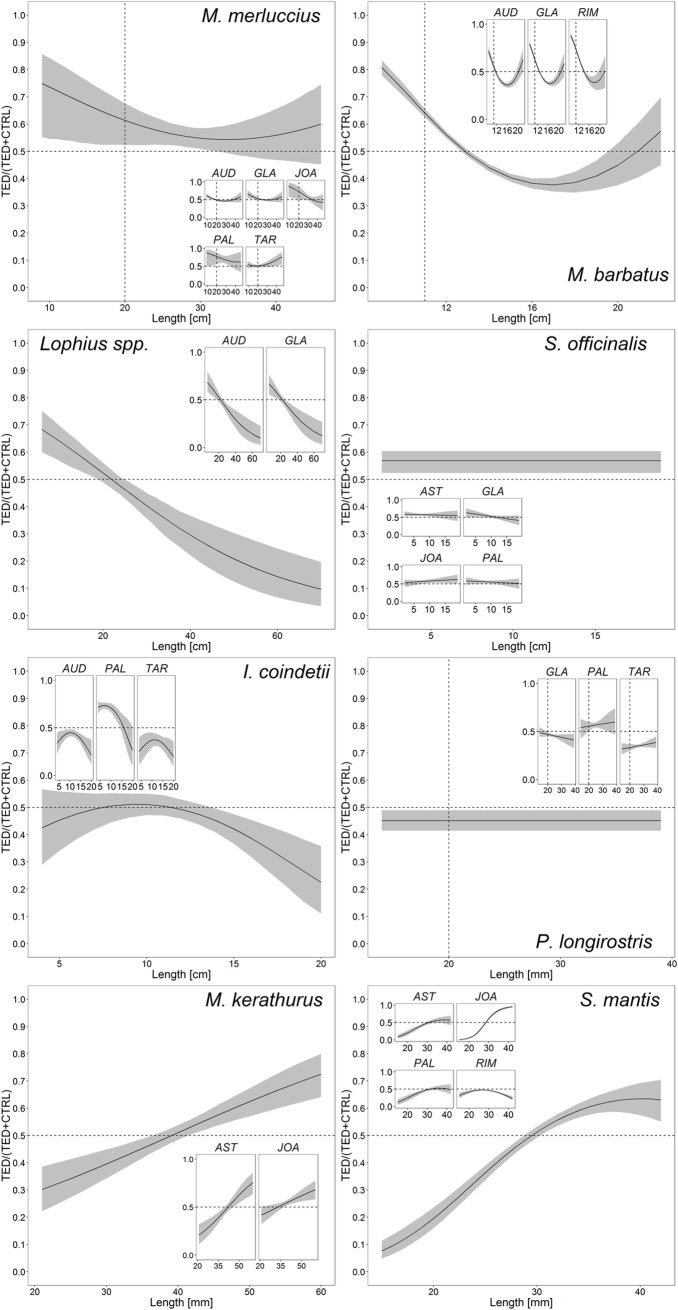
Proportions of the total catches of the TED nets according to the GLMM. The main diagrams show the model for all vessels, whereas the smallest diagrams show the model applied to each trawler. Interpretation: a value of 0.5 indicates an even catch between the TED and the CTRL nets, whereas a value of 0.25 indicates that the TED net caught 25% of the total fish of that length and the CTRL net caught 75%. Shaded area: 95% confidence interval.

The commercial species that accounted for more than 5% of the total weight of the catch of at least two vessels were selected for catch comparison analysis. They were *Lophius* spp., *Merluccius merluccius* (minimum landing size, MLS, 20 cm), *Mullus barbatus* (MLS, 11 cm), *Illex coindetii*, *Sepia officinalis*, *Melicerthus kerathurus*, *Parapenaeus longirostris* (MLS, 20 mm), and *Squilla mantis*. The LFDs of these species (Fig 4) differed among vessels, depending mostly on area, time of the year and fishing depth. Pooling of the individuals of each species (Fig 5) resulted in differences between TED and CTRL. The parameter estimates of the catch comparison models are reported in Table 5. The general trends of the proportion of individuals caught by the TED and CTRL nets are reported in Fig 6 together with the trends for each trawler (images from the videos showing small-sized species are shown in S1 Fig). For the three commercial fish species, the TED nets appeared to be more efficient than the CTRL nets in catching small individuals. Conversely, as TL increased the efficiency of the TED nets seemed to decrease, although for *M*. *merluccius* and *M*. *barbatus* the ratio was close to 0.5, indicating similar numbers of fish caught in the two nets. In the case of *Lophius* spp. increasing TL reduced the ratio, favouring the CTRL nets; however, the TL of the bulk of the catch was between 20 and 30 cm, the longest fish (> 30 cm) accounting for a small proportion of the total catch. Notably, their large head sometimes prevented the larger *Lophius* from going through the TED; at other times, medium to large *Lophius* individuals reached the TED not head first but transversely, on their flank (S2 Fig), and were crushed against the bars; in other cases they were pushed through the grid by the hydrodynamic force, and in other cases still they rolled up and swam through the opening on the upper side of the net. With regards to the molluscs, *S*. *officinalis* showed a constant slope with increasing size and a ratio that was always slightly greater than 0.5. *I*. *coindetii* showed a constant ratio close to 0.5 and a decreasing trend for larger individuals. The ratio for *P*. *longirostris* was slightly below 0.5. The TED nets caught fewer small individuals of *M*. *kerathurus* and *S*. *mantis* than the CTRL nets, but a greater proportion of larger individuals.

**Table 5:** GLMM parameter estimates of the catch selectivity logistic models. TED No. Ind.: TED number of individuals used for the analysis; CTRL No. Ind.: control number of individuals; SE: standard error.

Species	TED No. Ind.	CTRL No. Ind.	Model	Parameter	Estimate	SE	p
*Lophius* spp.	391	425	Linear	β_0_	1.02	0.31	<0.001
				β_1_	-0.05	0.01	<0.001
*M*. *merluccius*	2624	2478	Quadratic	β_0_	2.25	0.89	0.012
				β_1_	-0.12	0.04	0.004
				β_2_	0.002	0.0006	0.006
*M*. *barbatus*	5609	5257	Quadratic	β_0_	8.39	0.91	<0.001
				β_1_	-1.05	0.11	<0.001
				β_2_	0.03	0.004	<0.001
*I*. *coindetii*	1077	907	Quadratic	β_0_	-1.17	0.91	0.197
				β_1_	0.24	0.12	0.036
				β_2_	-0.013	0.005	0.013
*S*. *officinalis*	808	640	Constant	β_0_	0.34	0.07	<0.001
*S*. *mantis*	7716	9149	Quadratic	β_0_	-6.96	1.88	<0.001
				β_1_	0.38	0.08	<0.001
				β_2_	-0.005	0.001	<0.001
*P*. *longirostris*	4010	5191	Constant	β_0_	-0.20	0.22	0.362
*M*. *kerathurus*	948	881	Linear	β_0_	-1.65	0.67	0.014
				β_1_	0.02	2.92	0.004

Regarding bycatch, *Pteroplatytrygon violacea* (3 individuals caught by JOA, 1 by PAL) and *Dasyatis pastinaca* (38 individuals caught by AST) were the species of conservation interest, although their small number prevented statistical analysis. No turtles were caught at any time; however, several vessels fishing in the same areas at the same time as the trials reported incidentally catching some individuals.

## Discussion

To our knowledge, this is the first study comparing the catch efficiency of a traditional bottom trawl and of a trawl equipped with a flexible TED in routine operating conditions in the Mediterranean Sea.

Since the Adriatic bottom trawlers mainly catch juvenile and subadult sea turtles, population survival depends on the adoption of measures to avoid bycatch.

The flexible TED tested in this study was efficient, since it affected neither the weight nor the composition of the commercial catch; moreover, it significantly reduced debris and litter, resulting in greater catch quality through the exclusion of large objects that can damage the catch. These data are in line with those reported by Lucchetti et al. [23,24]. Recently, Strafella et al. [39] described the presence of several litter categories in the northern Adriatic Sea, the highest concentrations being found within 30 m depth of the surface and the lowest between 30 and 50 m. Similarly, in our study the highest litter concentrations were found at shallow and medium depth, although a detailed characterization of the litter size and composition is beyond the scope of this.

Differences in the catch rates observed during the trials among vessels depended mainly on the target species of the vessels, thus were influenced by the period of the year and both the area and depth. Notwithstanding, also during the same period at the same mean distance from the coast (as a proxy for depth) different vessels may have had different target species. This does not exclude that several other species may occur in the total catch (this is a multispecies fishery), and that was the reason of the 5% in weight cut off to choose the species to analyse for the catch comparison. This approach was in line with the main objective of the paper to assess the general performance of the TED whatever the season and the fishing conditions.

The LFDs of the main commercial species were similar in the TED and the CTRL nets, without significant size loss except for the larger individuals of *Lophius* spp. Common stingrays (*D*. *pastinaca*) and the pelagic stingrays (*P*. *violacea*) were found only in the CTRL nets. The data regarding *Lophius* spp., stingrays and large marine litter demonstrate the effectiveness of the TED in excluding large animals such as those vulnerable and/or endangered. The TED nets caught a smaller but not significantly different amount of *M*. *barbatus*, *M*. *merluccius* and *I*. *coindetii* compared with the CTRL nets. They also caught a significantly higher proportion of crustaceans, possibly as a result of the smaller amount of litter found in the TED nets, which can crush and destroy them [26]. These data therefore show that the TED is a valuable tool that can be fitted in traditional bottom trawls without compromising the catch of commercial species [23,24].

The study of Bycatch Reduction Devices (BRDs) and of strategies to mitigate the threat posed by fisheries is less advanced than the exploration of measures for the protection of sea turtle nesting sites (see Casale et al. [40] for a review of conservation measures). Measures to reduce sea turtle interactions with fisheries have been proposed in several papers for a number of fishing gears [40]. Those aimed at reducing the damage caused by pelagic longlines include circle hooks, which would be difficult to ingest, although findings are not conclusive [41]. With regards to the set nets, the only effective countermeasure to date are special lamps mounted on the net, which allow turtles to see the net and avoid being entangled [42,43]. Among the mitigation measures for bottom trawls tested to date (e.g., tow time restriction to avoid drowning), the TED seems to be effective. This is because fishers do not reduce their profit using TEDs while permitting the escape of endangered species [19]. The flexible TED evaluated by Lucchetti et al. [23] was less rigid than earlier designs [22,44], to provide the required stiffness and at the same time sufficient flexibility for winding around a standard winch; the latter feature also ensures that onboard procedures and instruments do not need to be altered and that net hauling is not lengthened.

Although the present data come from a pilot study, they do argue for a wider use of TEDs in the Adriatic and, hopefully, the Mediterranean Sea. According to the estimates of Lucchetti et al. [13], the adoption of this BRD may avoid the bycatch of more than 8,000 sea turtles a year in the Adriatic Sea and of more than 20,000 along the Italian coasts where bottom trawling is practiced; these captures are believed to result in 1,300 and 3,000 deaths a year, respectively. In Casale’s view [6], more than 39,000 sea turtles would avoid being caught incidentally in the entire Mediterranean, thus saving 7,500 individuals from death, although these figures may actually be underestimated [40]. The development of effective BRDs is thus an urgent task.

BRDs are not mandatory in any Mediterranean country and have been tested and promoted only on a voluntary basis or under economic incentives. A major obstacle is the fishers’s reluctance to modify their gears, which they fear would reduce profit or increase fuel consumption. Fishers’ compliance is clearly vital to reduce bycatch and depends heavily on the incentives [45]. If scientists can demonstrate that gear modifications do not affect commercial catches, most fishers would probably accept their use in case those modifications would become mandatory. Moreover, fishers should be involved to try to develop further TED modifications to make it more effective. There are examples worldwide that this strategy works [46]. The responses of the fishers involved in the sea trials in the Adriatic Sea were promising and suggest that their counterparts in the Mediterranean Sea might collaborate to the protection of sea turtles. Finally, the combination of education, outreach programs, and cooperative fisheries management provides a model of participatory bycatch assessment and ultimately bycatch mitigation [47]. Besides the technical innovations, other measures to reduce the impact of bycatch consist of raising fishers’ awareness and in training them in the best practices in sea turtle recovery after capture.

In conclusion, the conservation of sea turtles and other endangered species over a wide area such as the Mediterranean Sea is an environmental, technical and political challenge. Critically, although the measures involving gear modifications require investment, they are technically sound and capable of achieving conservation aims. While there was no conclusive data about the effects of TEDs on sea turtle bycatch, the present study serves to demonstrate that BRDs, such as the TED, can improve the catch quality and value. This is achieved by reducing the damage to the main commercial species (in particular crustaceans and small fishes) and the catch sorting time for fishers [26]. This pilot study demonstrates the need and value of further trials with the flexible TED in the Mediterranean Sea. Given the distribution and range of marine turtles, this research could extend to other countries in the region, in order to test and demonstrate broader effectiveness.

## Supporting information

S1 Table List of commercial species caught in the trials, mean CPUEW and standard error. (DOCX)

S2 Table List of discard species caught in the trials, mean CPUEW and standard error. (DOCX)

S3 Table List of debris types caught during the trials, mean CPUEW and standard error. Marine litter codes: A = PLASTIC: 01 = bottle; 02 = sheet; 03 = bag; 05 = fishing line (monofilament); 07 = synthetic rope; 08 = fishing net; 09 = cable ties; 10 = strapping band; 11 = crates and containers; 12 = mussel farming ropes; 13 = other. C = METAL: 01 = cans (food); 02 = cans (beverage); 03 = fishing related; 07 = cables; 08 = other. D = RUBBER: 01 = boots; 02 = balloons; 04 = tyre; 05 = glove; 06 = other. E = GLASS/CERAMIC: 01 = jar; 02 = bottle; 04 = other. F = NATURAL PRODUCTS: 01 = wood (processed); 02 = rope; 05 = other. G = MISCELLANEOUS: 01 = clothing/rags; 03 = other. DEBRIS: shells = empty shells; echinoderms = piece of sea urchins or dead sea urchins; wood = natural wood (branches or tree trunk); organic = unidentified organic material.
(DOCX)

S1 Fig Fish swimming in front of the TED and passing easily through its bars during trawling. (TIF)

S2 Fig Detail of an angler fish (*Lophius* spp.) (inside the red circle) blocked by the bars of the TED. This is probably to be ascribed to the large head of this species. In some cases, angler fish were pushed into the grid by the hydrodynamic force, in other cases they rolled until they reached the opening on the upper side of the net, before the TED. Although the TED in the picture is not the same design as the one used in the trials, the flexible bars exert the same effect on this species.
(TIF)
