# Supplementary material for: Bottom trawl catch comparison in the Mediterranean Sea: Flexible Turtle Excluder Device (TED) vs traditional gear
Source: PLoS One. 2019 Dec 4;14(12):e0216023. doi: 10.1371/journal.pone.0216023 (PMC6892479; doi:10.1371/journal.pone.0216023)
Supplement: S1 Table — (DOCX) [file pone.0216023.s001.docx]

**S1 Table: List of the commercial species caught during the trials with associated average CPUE_W_ and standard errors.**

| Species | AST TED | AST CTRL | AUD TED | AUD CTRL | GLA TED | GLA CTRL | JOA TED | JOA CTRL | PAL TED | PAL CTRL | RIM TED | RIM CTRL | TAR TED | TAR CTRL |
| --- | --- | --- | --- | --- | --- | --- | --- | --- | --- | --- | --- | --- | --- | --- |
| *Alloteuthis media* | 0.02 ± 0.002 | 0.15 ± 0.034 | 0.009 ± 0.001 | 0.028 ± 0.009 | 0.09 ± 0.015 | 0.09 ± 0.029 | 0.04 ± 0.01 | 0.03 ± 0.01 | 0.021 ± 0.006 | 0.02 ± 0.003 | 0.06 ± 0.015 | 0.04 ± 0.008 | 0.01 ± 0.003 | 0.02 ± 0.004 |
| *Arnoglossus laterna* | - | - | 0.013 ± 0.004 | 0.088 ± 0.05 | 0.08 ± 0.034 | 0.14 ± 0.065 | 0.14 ± 0.029 | 0.11 ± 0.039 | 0.134 ± 0.035 | 0.08 ± 0.031 | 0.46 ± 0.068 | 0.3 ± 0.03 | 0.11 ± 0.062 | 0.1 ± 0.028 |
| *Bolinus brandaris* | - | - | - | - | - | - | - | - | 5.123 ± 0.0 | 2.42 ± 0.981 | 0.61 ± 0.107 | 0.5 ± 0.065 | - | - |
| *Calappa granulata* | - | - | - | 0.139 ± 0.0 | - | - | - | - | - | - | - | - | - | - |
| *Cassidaria echinophora* | - | - | 0.097 ± 0.027 | 0.078 ± 0.023 | - | - | - | - | 0.118 ± 0.051 | 0.03 ± 0.009 | - | - | 0.13 ± 0.049 | 0.1 ± 0.03 |
| *Cepola macrophthalma* | - | - | 0.055 ± 0.0 | - | 0.07 ± 0.018 | 0.09 ± 0.028 | - | - | - | - | - | - | - | - |
| *Chelidonichthys lucernus* | 0.14 ± 0.093 | 0.06 ± 0.012 | 0.216 ± 0.035 | 0.259 ± 0.037 | 0.45 ± 0.053 | 0.52 ± 0.08 | 0.22 ± 0.047 | 0.37 ± 0.069 | 0.186 ± 0.035 | 0.17 ± 0.059 | 0.69 ± 0.147 | 0.5 ± 0.119 | 0.19 ± 0.039 | 0.14 ± 0.048 |
| *Citharus linguatula* | - | - | 0.227 ± 0.036 | 0.253 ± 0.047 | 0.33 ± 0.057 | 0.36 ± 0.064 | 0.19 ± 0.035 | 0.08 ± 0.0 | 0.172 ± 0.015 | 0.13 ± 0.064 | - | - | 0.07 ± 0.026 | 0.12 ± 0.085 |
| *Conger conger* | 0.54 ± 0.0 | 0.29 ± 0.0 | - | - | - | - | - | - | - | - | 0.28 ± 0.0 | 0.3 ± 0.0 | - | - |
| *Diplodus annularis* | 0.06 ± 0.012 | 0.05 ± 0.005 | - | - | - | - | - | - | - | - | - | 0.05 ± 0.011 | - | - |
| *Diplodus vulgaris* | - | - | 0.071 ± 0.0 | - | - | - | - | - | - | - | - | - | - | - |
| *Eledone cirrhosa* | - | - | 0.288 ± 0.07 | 0.421 ± 0.086 | 0.59 ± 0.0 | - | - | - | - | - | - | - | - | - |
| *Eledone moschata* | - | - | - | 0.54 ± 0.0 | - | - | - | - | - | - | - | - | - | - |
| *Eledone* spp | 0.3 ± 0.074 | - | - | - | 0.78 ± 0.128 | 1.24 ± 0.228 | 0.32 ± 0.085 | 0.12 ± 0.041 | 0.436 ± 0.125 | 0.33 ± 0.189 | 0.02 ± 0.002 | 0.03 ± 0.0 | 0.22 ± 0.053 | 0.91 ± 0.132 |
| *Eutrigla gurnardus* | - | - | 0.051 ± 0.01 | 0.032 ± 0.008 | 0.07 ± 0.021 | 0.13 ± 0.055 | 0.02 ± 0.0 | - | 0.021 ± 0.004 | 0.04 ± 0.0 | - | - | 0.04 ± 0.009 | 0.04 ± 0.014 |
| *Gobius niger* | - | - | - | 0.021 ± 0.0 | 0.01 ± 0.0 | - | 0.14 ± 0.023 | 0.12 ± 0.038 | 0.108 ± 0.031 | 0.14 ± 0.064 | 0.54 ± 0.084 | 0.5 ± 0.079 | 0.02 ± 0.0 | - |
| *Illex coindetii* | - | - | 0.857 ± 0.115 | 1.197 ± 0.154 | 0.37 ± 0.067 | 0.52 ± 0.081 | 0.09 ± 0.023 | - | 1.631 ± 0.476 | 1.01 ± 0.33 | 0.03 ± 0.0 | - | 0.56 ± 0.089 | 1.2 ± 0.132 |
| *Lepidorhombus boscii* | - | - | 0.057 ± 0.017 | - | - | - | - | - | - | - | - | 0.06 ± 0.005 | - | - |
| *Lepidotrigla cavillone* | - | - | 0.035 ± 0.0 | - | - | - | - | - | - | - | - | - | - | - |
| *Liocarcinus depurator* | - | - | 0.806 ± 0.156 | 1.28 ± 0.396 | - | - | - | 0.67 ± 0.132 | 0.76 ± 0.203 | 0.73 ± 0.263 | - | - | 1.35 ± 0.086 | 1.36 ± 0.271 |
| *Liza aurata* | 0.28 ± 0.073 | 0.11 ± 0.01 | - | - | - | - | - | - | - | - | - | - | - | - |
| *Loligo vulgaris* | 0.44 ± 0.161 | 0.57 ± 0.232 | 0.064 ± 0.016 | 0.075 ± 0.023 | 0.44 ± 0.064 | 0.4 ± 0.086 | 0.17 ± 0.027 | 0.17 ± 0.047 | 0.14 ± 0.037 | 0.14 ± 0.078 | 0.18 ± 0.05 | 0.07 ± 0.012 | 0.07 ± 0.018 | 0.11 ± 0.022 |
| *Lophius* spp | - | - | 1.344 ± 0.138 | 2.372 ± 0.46 | 2.03 ± 0.323 | 2.43 ± 0.495 | - | - | 0.694 ± 0.184 | 0.53 ± 0.232 | - | - | 0.2 ± 0.029 | 0.36 ± 0.122 |
| *Maja squinado* | - | - | - | - | 0.24 ± 0.006 | 0.49 ± 0.108 | - | - | - | - | - | - | - | - |
| *Melicertus kerathurus* | 0.92 ± 0.328 | 1.15 ± 0.44 | - | - | 0.21 ± 0.149 | 0.31 ± 0.244 | 1.69 ± 0.514 | 1.42 ± 0.615 | 0.057 ± 0.041 | 0.54 ± 0.065 | 1.13 ± 0.106 | 1 ± 0.211 | - | - |
| *Merlangius merlangus* | - | - | 0.239 ± 0.055 | 0.227 ± 0.06 | 0.2 ± 0.095 | 0.27 ± 0.134 | 3.85 ± 1.21 | 6.05 ± 1.309 | 0.019 ± 0.002 | - | 2.28 ± 0.368 | 2 ± 0.343 | 0.08 ± 0.035 | 0.04 ± 0.01 |
| *Merluccius merluccius* | - | - | 3.218 ± 0.221 | 3.887 ± 0.292 | 6.37 ± 0.597 | 6.44 ± 0.649 | 1.16 ± 0.157 | 1.26 ± 0.117 | 0.888 ± 0.343 | 0.37 ± 0.281 | 0.1 ± 0.062 | 0.05 ± 0.004 | 4.34 ± 0.335 | 3.58 ± 0.321 |
| *Microchirus ocellatus* | - | - | - | - | 0.03 ± 0.012 | 0.03 ± 0.006 | 0.03 ± 0.0 | - | - | - | - | - | - | - |
| *Microchirus variegatus* | - | - | 0.094 ± 0.025 | 0.033 ± 0.01 | 0.12 ± 0.031 | 0.14 ± 0.058 | 0.02 ± 0.004 | - | - | - | 0.01 ± 0.0 | 0.009 ± 0.0 | - | - |
| *Micromesistius poutassou* | - | - | 0.147 ± 0.0 | 0.063 ± 0.0 | - | - | - | - | 0.047 ± 0.0 | - | - | - | - | - |
| *Mullus barbatus* | 0.04 ± 0.0 | - | 1.54 ± 0.367 | 1.766 ± 0.478 | 4.44 ± 0.985 | 4.9 ± 1.109 | 0.7 ± 0.197 | 0.2 ± 0.105 | 0.219 ± 0.043 | 0.15 ± 0.02 | 4.23 ± 0.621 | 3 ± 0.361 | 0.16 ± 0.036 | 0.1 ± 0.03 |
| *Mullus surmuletus* | - | - | 0.067 ± 0.014 | 0.067 ± 0.01 | 0.06 ± 0.016 | 0.08 ± 0.019 | - | - | - | - | 0.08 ± 0.025 | - | - | 0.05 ± 0.0 |
| *Mustelus mustelus* | 1.84 ± 0.313 | 6.1 ± 2.705 | - | - | - | - | - | - | - | - | - | - | - | - |
| *Nephrops norvegicus* | - | - | 0.355 ± 0.057 | 0.381 ± 0.064 | 0.27 ± 0.091 | 0.22 ± 0.065 | 0.36 ± 0.118 | 0.17 ± 0.057 | 0.147 ± 0.072 | 0.12 ± 0.01 | - | - | 0.12 ± 0.05 | 0.24 ± 0.097 |
| *Octopus vulgaris* | - | - | 2.237 ± 1.335 | 1.044 ± 0.241 | 1.86 ± 0.0 | - | - | 0.96 ± 0.0 | - | 0.07 ± 0.0 | - | - | 0.87 ± 0.283 | 2.14 ± 0.922 |
| *Pagellus acarne* | - | - | 0.132 ± 0.0 | 0.057 ± 0.016 | 0.07 ± 0.013 | - | - | - | - | - | - | - | - | - |
| *Pagellus erythrinus* | 0.28 ± 0.053 | 0.09 ± 0.035 | 0.069 ± 0.023 | 0.092 ± 0.062 | 0.17 ± 0.065 | 0.27 ± 0.069 | - | - | 0.022 ± 0.003 | 0.06 ± 0.0 | - | - | 0.04 ± 0.007 | 0.05 ± 0.0 |
| *Pagrus pagrus* | - | - | - | - | - | - | - | - | - | - | - | - | 0.07 ± 0.0 | - |
| *Palinurus elephas* | - | - | - | - | - | 0.5 ± 0.0 | - | - | - | - | - | - | - | - |
| *Parapenaeus longirostris* | - | - | 0.377 ± 0.084 | 0.353 ± 0.101 | 1.32 ± 0.403 | 1.97 ± 0.538 | - | - | 0.727 ± 0.262 | 0.89 ± 0.31 | - | - | 1.33 ± 0.219 | 2.5 ± 0.555 |
| *Phycis blennoides* | - | - | 0.03 ± 0.005 | 0.014 ± 0.002 | - | - | - | 0.14 ± 0.0 | - | - | - | - | - | - |
| *Phycis* spp | - | - | - | 0.017 ± 0.001 | - | - | - | - | - | - | - | - | - | - |
| *Platichthys flesus* | 0.26 ± 0.101 | 0.31 ± 0.188 | - | - | - | - | - | - | - | - | - | - | 0.19 ± 0.016 | - |
| *Pomatomus saltator* | - | - | - | - | 0.72 ± 0.0 | - | - | - | - | - | 0.7 ± 0.171 | 1 ± 0.449 | - | - |
| *Psetta maxima* | - | - | - | 0.196 ± 0.0 | - | - | 0.29 ± 0.0 | - | - | - | 0.25 ± 0.049 | 0.3 ± 0.145 | - | - |
| *Raja asterias* | - | - | 0.351 ± 0.035 | 0.941 ± 0.202 | 0.3 ± 0.108 | 0.75 ± 0.195 | 0.29 ± 0.0 | - | 1.058 ± 0.315 | 0.45 ± 0.13 | - | - | 0.89 ± 0.215 | 0.65 ± 0.191 |
| *Raja clavata* | - | - | - | - | 0.32 ± 0.0 | 1.03 ± 0.314 | - | - | - | - | - | - | - | - |
| *Raja miraletus* | - | - | 0.15 ± 0.0 | 0.389 ± 0.0 | - | - | - | - | - | - | - | - | - | - |
| *Sciaena umbra* | - | - | - | - | - | - | - | - | - | - | - | 0.3 ± 0.0 | - | - |
| *Scomber japonicus* | - | - | 0.092 ± 0.011 | 0.283 ± 0.057 | - | - | - | - | - | - | - | - | 0.08 ± 0.0 | - |
| *Scomber scombrus* | - | - | 0.067 ± 0.016 | - | 0.3 ± 0.14 | 0.62 ± 0.391 | 0.23 ± 0.059 | 0.43 ± 0.124 | - | 0.06 ± 0.003 | 0.51 ± 0.294 | 0.2 ± 0.045 | 0.47 ± 0.206 | 0.36 ± 0.078 |
| *Scophthalmus rhombus* | 0.33 ± 0.081 | 0.38 ± 0.039 | 0.237 ± 0.083 | 0.169 ± 0.027 | 0.18 ± 0.025 | 0.55 ± 0.0 | - | - | - | 0.2 ± 0.0 | - | - | 0.15 ± 0.0 | - |
| *Scorpaena notata* | - | - | 0.089 ± 0.049 | 0.108 ± 0.067 | 0.07 ± 0.016 | 0.11 ± 0.026 | 0.04 ± 0.009 | 0.05 ± 0.021 | 0.029 ± 0.0 | - | - | - | - | - |
| *Scorpaena porcus* | 0.35 ± 0.0 | 0.27 ± 0.0 | - | - | 0.25 ± 0.0 | - | - | - | - | - | - | - | 0.09 ± 0.042 | 0.04 ± 0.0 |
| *Scorpaena scrofa* | - | - | 0.111 ± 0.035 | 0.191 ± 0.088 | - | - | - | - | - | - | - | - | - | - |
| *Scyliorhinus canicula* | - | - | 0.058 ± 0.017 | 0.132 ± 0.021 | - | 0.16 ± 0.018 | - | - | - | - | - | - | - | - |
| *Sepia elegans* | - | - | 0.03 ± 0.008 | 0.039 ± 0.011 | - | - | 0.03 ± 0.008 | 0.04 ± 0.007 | - | - | - | - | - | - |
| *Sepia officinalis* | 3.09 ± 0.414 | 2.46 ± 0.276 | 0.036 ± 0.002 | 0.085 ± 0.031 | 2.38 ± 0.421 | 2.79 ± 0.557 | 1.63 ± 0.296 | 0.95 ± 0.238 | 2.08 ± 0.627 | 1.46 ± 0.2 | 2.08 ± 0.622 | 0.9 ± 0.209 | 0.1 ± 0.0 | - |
| *Sepiola rondeleti* | - | - | 0.011 ± 0.002 | 0.011 ± 0.002 | 0.32 ± 0.03 | 0.23 ± 0.031 | - | - | - | - | - | - | - | - |
| *Solea impar* | - | - | - | - | - | - | - | - | 0.006 ± 0.0 | - | - | - | - | - |
| *Solea solea* | 3.86 ± 0.857 | 1.71 ± 0.657 | 0.206 ± 0.035 | 0.148 ± 0.033 | 0.15 ± 0.054 | 0.2 ± 0.028 | 0.54 ± 0.115 | 0.17 ± 0.045 | 0.224 ± 0.057 | 0.17 ± 0.026 | 0.71 ± 0.104 | 1 ± 0.203 | 0.26 ± 0.1 | 0.21 ± 0.078 |
| *Sparus aurata* | 0.23 ± 0.099 | 0.32 ± 0.057 | - | - | 0.14 ± 0.038 | 0.14 ± 0.063 | - | 0.04 ± 0.0 | - | - | - | 0.1 ± 0.0 | - | - |
| *Sphyraena sphyraena* | - | - | - | - | - | 0.05 ± 0.0 | - | - | - | - | 0.09 ± 0.0 | 0.3 ± 0.098 | - | - |
| *Spondilyosoma cantharus* | 0.19 ± 0.0 | - | - | 0.066 ± 0.0 | - | - | - | - | - | - | - | - | - | - |
| *Squalus acanthias* | - | - | 0.022 ± 0.0 | - | - | - | 1 ± 0.139 | - | - | - | - | - | - | - |
| *Squilla mantis* | 0.99 ± 0.306 | 1.53 ± 0.473 | 0.027 ± 0.004 | 0.054 ± 0.036 | 0.42 ± 0.271 | 0.73 ± 0.539 | 1.85 ± 0.55 | 2.33 ± 1.016 | 1.072 ± 0.357 | 0.92 ± 0.347 | 26.25 ± 2.248 | 30 ± 3.01 | 0.53 ± 0.414 | 0.41 ± 0.346 |
| *Torpedo marmorata* | - | - | 0.117 ± 0.001 | - | - | - | - | - | - | - | 0.33 ± 0.0 | - | - | - |
| *Trachinus draco* | 0.23 ± 0.108 | 0.08 ± 0.0 | 0.203 ± 0.055 | 0.17 ± 0.041 | 0.28 ± 0.048 | 0.23 ± 0.04 | 0.04 ± 0.0 | - | 0.114 ± 0.045 | 0.14 ± 0.075 | - | - | 0.2 ± 0.066 | 0.12 ± 0.029 |
| *Trachurus mediterraneus* | - | - | 0.107 ± 0.0 | 0.008 ± 0.0 | - | 0.03 ± 0.0 | - | 0.03 ± 0.003 | - | - | 0.05 ± 0.005 | - | - | - |
| *Trachurus* spp | 0.25 ± 0.069 | 0.23 ± 0.036 | - | 0.031 ± 0.0 | 0.03 ± 0.0 | 0.09 ± 0.036 | 0.03 ± 0.0 | - | 0.076 ± 0.044 | 0.03 ± 0.0 | - | - | 0.19 ± 0.041 | 0.21 ± 0.037 |
| *Trachurus trachurus* | - | - | 0.362 ± 0.235 | 0.136 ± 0.085 | - | - | - | - | - | - | - | - | - | - |
| *Trigloporus lastoviza* | - | - | - | 0.041 ± 0.0 | - | - | - | - | - | - | - | - | - | - |
| *Trisopterus minutus capelanus* | - | - | 0.167 ± 0.021 | 0.274 ± 0.03 | 1.29 ± 0.192 | 1.75 ± 0.301 | 0.15 ± 0.034 | 0.23 ± 0.068 | 0.477 ± 0.068 | 0.32 ± 0.161 | - | - | 0.31 ± 0.039 | 0.19 ± 0.034 |
| *Uranoscopus scaber* | - | - | 0.101 ± 0.019 | 0.151 ± 0.031 | 0.11 ± 0.012 | 0.1 ± 0.018 | - | - | 0.166 ± 0.051 | 0.2 ± 0.057 | - | - | 0.09 ± 0.033 | 0.07 ± 0.023 |
| *Zeus faber* | 0.18 ± 0.0 | - | 0.312 ± 0.055 | 0.212 ± 0.0 | 0.19 ± 0.046 | 0.3 ± 0.088 | - | 0.33 ± 0.136 | 0.099 ± 0.0 | 0.21 ± 0.0 | 0.12 ± 0.022 | 0.2 ± 0.037 | - | 0.05 ± 0.0 |
