# Supplementary material for: Bottom trawl catch comparison in the Mediterranean Sea: Flexible Turtle Excluder Device (TED) vs traditional gear
Source: PLoS One. 2019 Dec 4;14(12):e0216023. doi: 10.1371/journal.pone.0216023 (PMC6892479; doi:10.1371/journal.pone.0216023)
Supplement: S2 Table — (DOCX) [file pone.0216023.s002.docx]

**S2 Table: List of the discard species caught during the trials with associated average CPUE_W_ and standard errors.**

| Species | AST TED | AST CTRL | AUD TED | AUD CTRL | GLA TED | GLA CTRL | JOA TED | JOA CTRL | PAL TED | PAL CTRL | RIM TED | RIM CTRL | TAR TED | TAR CTRL |
| --- | --- | --- | --- | --- | --- | --- | --- | --- | --- | --- | --- | --- | --- | --- |
| *Acanthocardia aculeata* | - | - | 0.033 ± 0.0 | 0.046 ± 0.018 | - | - | - | - | - | - | - | - | - | - |
| *Actiniaria* spp | 0.66 ± 0.39 | 6.22 ± 2.6 | - | - | - | 0.578 ± 0.0 | 0.33 ± 0.0 | - | - | - | - | - | - | - |
| *Aegaeon cataphractus* | - | - | 0.013 ± 0.007 | 0.017 ± 0.008 | 0.028 ± 0.003 | 0.006 ± 0.001 | - | - | - | - | - | - | 0.03 ± 0.0 | - |
| *Alcyonium palmatum* | - | - | 0.063 ± 0.02 | 0.131 ± 0.034 | 1.456 ± 0.575 | 1.259 ± 0.625 | - | 0.02 ± 0.0 | - | - | - | - | - | - |
| *Alloteuthis media* | 0.08 ± 0.0 | 0.33 ± 0.19 | 0.031 ± 0.009 | 0.043 ± 0.016 | 0.066 ± 0.018 | 0.143 ± 0.068 | 0.34 ± 0.0 | 0.15 ± 0.03 | 0.26 ± 0.071 | 0.469 ± 0.183 | 0.085 ± 0.03 | 0.07 ± 0.02 | 0.08 ± 0.031 | - |
| *Alosa fallax* | - | - | - | - | - | - | - | - | - | - | 0.513 ± 0.07 | 1.82 ± 0.8 | - | - |
| *Alpheus dentipes* | - | - | - | - | - | - | - | - | 0.01 ± 0.0 | 0.017 ± 0.006 | - | - | - | - |
| *Alpheus glaber* | - | - | - | - | - | - | - | - | - | - | - | - | 0.01 ± 0.009 | 0.005 ± 0.0 |
| *Anadara demiri* | - | - | - | - | - | - | - | - | 0.06 ± 0.0 | 0.164 ± 0.094 | 1.391 ± 0.5 | 0.95 ± 0.4 | - | - |
| *Anadara kagoshimensis* | - | - | - | - | - | - | - | - | - | - | 0.045 ± 0.0 | 0.16 ± 0.02 | - | - |
| *Antonogadus megalokynodon* | - | - | 0.055 ± 0.026 | 0.037 ± 0.007 | 0.207 ± 0.077 | 0.24 ± 0.122 | - | 0.05 ± 0.0 | - | - | - | - | 0.1 ± 0.038 | 0.072 ± 0.022 |
| *Aphrodite aculeata* | 1.18 ± 0.39 | 0.75 ± 0.29 | - | - | 1.212 ± 0.5 | 1.08 ± 0.406 | - | 0.39 ± 0.0 | - | - | - | - | - | - |
| *Aplisia* spp | 1 ± 0.0 | 1.39 ± 0.0 | 0.142 ± 0.082 | 0.246 ± 0.209 | - | - | - | 0.39 ± 0.16 | - | - | - | - | - | - |
| *Aplysiidae* spp | - | - | - | - | - | - | 0.48 ± 0.0 | - | - | - | - | - | - | - |
| *Aporrhais pespelecani* | 0.28 ± 0.0 | - | 0.091 ± 0.014 | 0.175 ± 0.035 | - | - | 0.81 ± 0.22 | 0.68 ± 0.25 | 0.59 ± 0.223 | 4.167 ± 1.945 | 0.275 ± 0.09 | 0.14 ± 0.05 | 0.5 ± 0.281 | 0.377 ± 0.115 |
| *Armina tigrina* | - | - | - | - | 0.087 ± 0.0 | - | - | - | - | - | - | - | 0.03 ± 0.0 | - |
| *Arnoglossus laterna* | 2.45 ± 1.09 | - | 0.052 ± 0.013 | 0.105 ± 0.02 | 0.165 ± 0.025 | 0.221 ± 0.048 | 1.1 ± 0.03 | 0.55 ± 0.11 | 0.13 ± 0.032 | 0.229 ± 0.028 | 0.286 ± 0.08 | 0.11 ± 0.01 | 0.6 ± 0.174 | 0.472 ± 0.222 |
| *Arnoglossus thori* | - | - | - | - | 0.497 ± 0.396 | 0.237 ± 0.06 | - | - | - | - | - | - | - | - |
| *Ascidia virginia* | - | - | - | - | - | - | - | - | - | - | 0.007 ± 0.0 | - | - | - |
| *Ascidiaceae* spp | 13.22 ± 4.64 | 15.3 ± 9.66 | - | - | - | - | - | 0.37 ± 0.25 | - | - | - | - | - | - |
| *Astropecten irregularis* | 0.92 ± 0.21 | 2.1 ± 1.34 | 0.246 ± 0.087 | 0.44 ± 0.16 | 0.708 ± 0.352 | 0.699 ± 0.348 | - | 0.3 ± 0.07 | 0.26 ± 0.11 | 0.555 ± 0.098 | 0.516 ± 0.2 | 0.26 ± 0.06 | 0.1 ± 0.045 | 0.097 ± 0.071 |
| *Atrina pectinata* | - | - | - | - | - | - | - | 79.02 ± 49.3 | - | - | - | - | - | - |
| *Atrina truncata* | - | - | - | 0.799 ± 0.0 | - | - | - | - | - | - | - | 0.35 ± 0.0 | - | - |
| *Balistes capriscus* | - | - | - | - | - | - | - | - | - | - | 0.046 ± 0.0 | - | - | - |
| *Blennius ocellaris* | - | - | 0.158 ± 0.049 | 0.13 ± 0.058 | 0.218 ± 0.071 | 0.137 ± 0.024 | - | - | 0.11 ± 0.014 | 0.136 ± 0.0 | - | - | - | - |
| *Bolinus brandaris* | 3.26 ± 1.05 | 2.53 ± 0.83 | 0.019 ± 0.005 | 0.06 ± 0.022 | - | 0.183 ± 0.0 | 0.14 ± 0.0 | - | 0.52 ± 0.205 | 0.739 ± 0.25 | 0.328 ± 0.1 | 0.47 ± 0.1 | 0.4 ± 0.193 | 0.214 ± 0.0 |
| *Boops boops* | - | - | 0.33 ± 0.196 | 0.379 ± 0.091 | - | 0.192 ± 0.087 | - | - | 0.3 ± 0.129 | 0.705 ± 0.0 | 0.13 ± 0.1 | 1.18 ± 0.3 | 0.5 ± 0.0 | - |
| *Brachinotus gemellari* | - | - | - | - | - | - | - | - | - | - | - | 0.1 ± 0.06 | - | - |
| *Buglossidium luteum* | - | - | - | - | 0.123 ± 0.0 | 0.657 ± 0.38 | - | - | - | 0.178 ± 0.0 | - | - | 0.1 ± 0.0 | 0.09 ± 0.0 |
| *Calappa granulata* | - | - | 0.093 ± 0.041 | 0.04 ± 0.008 | - | - | - | - | - | - | - | - | - | - |
| *Calliactis parasitica* | 3.95 ± 1.1 | 1.46 ± 0.29 | 0.012 ± 0.004 | 0.058 ± 0.046 | - | - | - | - | - | - | - | - | - | - |
| *Callionymus maculatus* | - | - | 0.044 ± 0.008 | 0.065 ± 0.013 | 0.096 ± 0.023 | 0.14 ± 0.039 | - | - | 0.05 ± 0.029 | - | - | - | 0.1 ± 0.025 | 0.217 ± 0.045 |
| *Calliostoma granulatum* | - | - | - | - | 0.1 ± 0.0 | 0.05 ± 0.0 | - | - | - | - | - | - | - | - |
| *Capsule ovigere elasmobranchi* | - | - | 0.074 ± 0.03 | 0.057 ± 0.016 | 0.084 ± 0.013 | 0.121 ± 0.032 | - | - | 0.19 ± 0.033 | 0.155 ± 0.034 | - | - | 0.2 ± 0.041 | 0.25 ± 0.052 |
| *Carcinus aestuarii* | 0.92 ± 0.25 | 1.4 ± 0.32 | - | - | - | - | - | - | - | - | - | - | - | - |
| *Cassidaria echinophora* | - | - | 0.161 ± 0.058 | 0.402 ± 0.0 | - | - | - | - | - | - | - | - | - | - |
| *Centrolophus niger* | - | - | - | - | - | - | - | 0.42 ± 0.0 | - | - | - | - | - | - |
| *Cepola macrophthalma* | - | - | 0.088 ± 0.015 | 0.04 ± 0.014 | 0.165 ± 0.035 | 0.23 ± 0.059 | 0.29 ± 0.0 | 0.14 ± 0.0 | 0.09 ± 0.023 | 0.127 ± 0.035 | - | - | 0.1 ± 0.056 | 0.265 ± 0.0 |
| *Chelidonichthys lucernus* | 1.63 ± 0.54 | 1.02 ± 0.17 | 0.125 ± 0.0 | 0.595 ± 0.222 | - | 0.334 ± 0.0 | - | - | - | - | - | - | 1 ± 0.175 | 0.578 ± 0.123 |
| *Chlamys opercularis* | 0.13 ± 0.0 | 13.98 ± 11.83 | - | - | 0.04 ± 0.023 | - | - | - | - | 0.02 ± 0.0 | - | - | - | - |
| *Chlamys varia* | 9.61 ± 2.25 | 2.55 ± 0.0 | - | - | - | - | - | - | - | - | - | - | - | - |
| *Cirolana* spp | - | - | - | - | - | - | - | - | - | - | - | 0.01 ± 0.0003 | - | - |
| *Citharus linguatula* | - | - | 0.166 ± 0.094 | 0.119 ± 0.037 | 0.452 ± 0.13 | 0.504 ± 0.111 | - | - | - | - | 0.207 ± 0.0 | - | 0.03 ± 0.0 | - |
| *Conger conger* | - | - | 0.075 ± 0.019 | 0.759 ± 0.484 | - | - | - | - | - | - | 0.301 ± 0.0 | - | 1 ± 0.0 | 0.519 ± 0.0 |
| *Corbula gibba* | - | - | - | - | - | - | - | - | - | 0.016 ± 0.0 | - | - | - | - |
| *Corystes cassivelaunus* | - | - | - | - | - | - | - | - | - | 0.022 ± 0.0 | - | - | - | - |
| *Cuspidaria cuspidata* | - | - | - | - | - | - | - | - | - | - | - | - | - | 0.014 ± 0.0 |
| *Cymodocea nodosa* | - | - | - | 0.009 ± 0.0 | - | - | - | - | - | - | - | - | - | - |
| *CYMOTHOIDAE NDD* | - | - | 0.007 ± 0.0 | - | - | - | - | - | - | - | - | - | - | - |
| *Dardanus arrosor* | - | - | - | - | 0.699 ± 0.131 | 0.832 ± 0.0 | - | - | - | - | - | - | - | - |
| *Debris conchiglie* | - | - | - | - | - | 1.889 ± 0.0 | - | - | - | - | - | - | - | - |
| *Diodora italica* | 0.2 ± 0.05 | - | 0.172 ± 0.0 | - | - | - | - | - | - | - | - | - | - | - |
| *Diplodus annularis* | - | - | - | - | - | 0.435 ± 0.0 | - | - | 0.7 ± 0.0 | - | 0.16 ± 0.0 | - | - | - |
| *Echinaster sepositus* | - | - | 0.032 ± 0.025 | 0.04 ± 0.015 | - | 0.17 ± 0.087 | - | - | - | - | - | - | - | - |
| *Echinocardium cordatum* | - | - | - | - | - | - | - | 0.47 ± 0.15 | - | - | - | - | - | - |
| *Eledone cirrhosa* | - | - | 0.06 ± 0.0 | - | - | - | - | - | - | - | - | - | - | - |
| *Eledone* spp | - | - | - | - | - | - | - | - | - | - | - | - | 0.2 ± 0.023 | 0.12 ± 0.0 |
| *Engraulis encrasicolus* | 0.5 ± 0.0 | - | 0.567 ± 0.192 | 0.798 ± 0.42 | 0.159 ± 0.077 | - | 0.4 ± 0.0 | 0.19 ± 0.04 | 0.18 ± 0.059 | 0.254 ± 0.134 | 1.374 ± 0.7 | 0.36 ± 0.09 | 0.7 ± 0.303 | 0.636 ± 0.37 |
| *Epitonium commune* | - | - | - | - | - | - | - | - | - | - | - | - | 0.04 ± 0.002 | 0.026 ± 0.003 |
| *Ethusa mascarone* | 0.22 ± 0.08 | 0.02 ± 0.0 | - | - | - | - | - | - | - | - | - | - | - | - |
| *Eurynome aspera* | 0.13 ± 0.0 | - | - | - | - | - | - | - | - | - | - | - | - | - |
| *Eutrigla gurnardus* | - | - | 0.333 ± 0.047 | 0.427 ± 0.083 | 0.985 ± 0.37 | 0.454 ± 0.09 | - | - | 0.12 ± 0.013 | 0.579 ± 0.0 | - | - | 0.3 ± 0.06 | 0.245 ± 0.089 |
| *Flexopecten glaber* | - | - | 0.02 ± 0.0 | - | - | - | - | - | - | - | 0.014 ± 0.0 | 0.02 ± 0.005 | - | - |
| *Funiculina quadrangularis* | - | - | 0.117 ± 0.088 | 0.063 ± 0.013 | 0.117 ± 0.034 | 0.331 ± 0.163 | - | - | 0.05 ± 0.03 | 0.011 ± 0.003 | - | - | - | - |
| *Glossus humanus* | - | - | - | - | - | - | - | 1.06 ± 0.0 | - | - | - | - | - | - |
| *Gobius niger* | - | 0.22 ± 0.0 | - | - | - | - | 0.42 ± 0.0 | 0.24 ± 0.07 | 0.18 ± 0.041 | - | 0.209 ± 0.05 | 0.17 ± 0.03 | 0.4 ± 0.024 | 0.369 ± 0.176 |
| *Gobius* spp | - | - | - | - | - | - | - | - | 0.08 ± 0.065 | 0.133 ± 0.064 | - | - | - | - |
| *Goneplax rhomboides* | 1.05 ± 0.0 | - | 0.015 ± 0.003 | 0.025 ± 0.007 | 0.035 ± 0.007 | 0.083 ± 0.02 | 1.38 ± 0.72 | 0.17 ± 0.1 | 0.19 ± 0.059 | 0.273 ± 0.061 | 0.131 ± 0.01 | 0.18 ± 0.1 | 0.1 ± 0.033 | 0.098 ± 0.04 |
| *Hexaplex trunculus* | 7.04 ± 2.79 | 11.26 ± 4.65 | - | - | - | - | - | - | - | - | 0.517 ± 0.1 | 0.76 ± 0.2 | - | - |
| *Hiatella arctica* | - | - | - | - | - | - | - | - | - | - | - | - | 0.002 ± 0.0 | - |
| *HOLOTHURIIDAE NDD* | - | - | 0.261 ± 0.066 | 0.455 ± 0.134 | - | - | - | - | - | - | - | - | - | - |
| *Holoturia forksali* | 7.85 ± 4.35 | 6.21 ± 1.14 | - | 0.301 ± 0.0 | 0.592 ± 0.073 | 0.726 ± 0.415 | - | 0.9 ± 0.0 | - | - | - | - | - | - |
| *Ilia nucleus* | 0.23 ± 0.13 | 1.74 ± 1.72 | - | - | - | - | - | - | - | - | - | - | - | - |
| *Illex coindetii* | - | - | 0.184 ± 0.065 | 0.185 ± 0.073 | 0.176 ± 0.079 | 0.269 ± 0.048 | - | - | - | 0.213 ± 0.071 | - | - | - | - |
| *Inachus comunissimus* | 0.24 ± 0.09 | 0.19 ± 0.17 | 0.004 ± 0.001 | 0.017 ± 0.006 | - | - | - | - | - | - | - | - | - | 0.013 ± 0.0 |
| *Inachus dorsettensis* | 0.52 ± 0.24 | 0.18 ± 0.0 | - | 0.01 ± 0.004 | - | - | - | - | - | - | - | - | - | - |
| *Laevicardium oblongum* | 0.99 ± 0.69 | 0.03 ± 0.0 | - | - | - | - | - | - | - | - | - | - | - | - |
| *Lepidotrigla cavillone* | - | - | - | - | 0.455 ± 0.081 | 0.461 ± 0.113 | - | - | 0.08 ± 0.0 | 0.045 ± 0.0 | - | - | 0.06 ± 0.0 | 0.171 ± 0.0 |
| *Lesuerigobius friesii* | - | - | 0.01 ± 0.003 | 0.019 ± 0.006 | 0.008 ± 0.0 | 0.039 ± 0.024 | 0.15 ± 0.04 | 0.04 ± 0.01 | - | - | 0.018 ± 0.0 | - | 0.1 ± 0.029 | 0.042 ± 0.007 |
| *Lima hians* | 0.3 ± 0.08 | - | - | - | - | - | - | - | - | - | - | - | - | - |
| *Liocarcinus corrugatus* | - | - | - | - | - | - | - | - | 3.43 ± 0.0 | - | - | - | - | - |
| *Liocarcinus depurator* | - | 0.15 ± 0.0 | 4.569 ± 0.907 | 4.945 ± 1.009 | 5.534 ± 1.404 | 6.91 ± 1.875 | 23.02 ± 0.62 | 5.74 ± 1.03 | 1.5 ± 0.255 | 2.145 ± 0.739 | 0.139 ± 0.03 | 0.07 ± 0.02 | 10 ± 1.119 | 6.929 ± 1.255 |
| *Liocarcinus vernalis* | - | - | - | - | - | - | - | - | - | - | 0.268 ± 0.0 | - | - | - |
| *Loligo forbesi* | - | - | - | - | - | - | - | - | 0.08 ± 0.0 | - | - | - | - | - |
| *Loligo vulgaris* | - | 0.24 ± 0.04 | 0.082 ± 0.0 | - | 0.108 ± 0.0 | - | - | - | - | - | - | - | - | - |
| *Lophius* spp | - | - | 0.092 ± 0.017 | 0.189 ± 0.041 | - | - | - | - | 0.04 ± 0.013 | 0.03 ± 0.012 | - | - | 0.8 ± 0.191 | 0.578 ± 0.106 |
| *Macropodia longirostris* | 0.07 ± 0.0 | - | - | - | - | - | - | - | - | - | - | - | - | - |
| *Marthasterias glacialis* | - | - | 0.061 ± 0.027 | 0.543 ± 0.0 | 2.07 ± 0.585 | 3.058 ± 0.649 | - | - | - | - | - | - | - | - |
| *Medorippe lanata* | 1.87 ± 0.51 | 1.76 ± 0.58 | 0.013 ± 0.004 | 0.037 ± 0.012 | 0.13 ± 0.0 | 0.081 ± 0.0 | 0.21 ± 0.0 | 0.16 ± 0.05 | 0.23 ± 0.07 | 0.177 ± 0.058 | 0.29 ± 0.09 | 0.18 ± 0.07 | 0.3 ± 0.0 | - |
| *Melicertus kerathurus* | - | - | - | - | - | - | - | 0.11 ± 0.04 | - | - | 0.172 ± 0.007 | 0.15 ± 0.04 | - | - |
| *Merlangius merlangus* | - | - | 0.22 ± 0.038 | 0.282 ± 0.068 | - | - | - | 0.31 ± 0.07 | 0.04 ± 0.007 | - | 0.128 ± 0.0 | 0.3 ± 0.0 | - | 0.093 ± 0.0 |
| *Merluccius merluccius* | - | - | 0.136 ± 0.026 | 0.348 ± 0.074 | 0.395 ± 0.107 | 0.677 ± 0.243 | - | - | 0.14 ± 0.088 | 0.296 ± 0.133 | - | - | 1 ± 0.088 | 1.25 ± 0.252 |
| *Microchirus variegatus* | 0.31 ± 0.0 | 0.5 ± 0.0 | 0.13 ± 0.0 | 0.133 ± 0.054 | - | - | 0.22 ± 0.11 | 0.09 ± 0.0 | - | - | - | - | - | - |
| *Microcosmus sulcatus* | - | - | - | - | - | - | - | - | - | - | - | - | 0.4 ± 0.0 | - |
| *Modiolarca subpicta* | - | - | - | - | 0.023 ± 0.0 | 0.007 ± 0.0 | - | - | - | - | - | - | 0.06 ± 0.0 | - |
| *Modiolus barbatus* | 1.12 ± 0.0 | - | - | - | - | - | - | - | - | - | - | - | - | - |
| *Monia patelliformis* | - | - | - | - | - | 0.165 ± 0.0 | - | - | - | - | - | - | - | - |
| *Mullus barbatus* | - | 0.55 ± 0.0 | 0.146 ± 0.084 | 0.178 ± 0.062 | 1.945 ± 0.585 | 4.278 ± 1.168 | - | - | 0.18 ± 0.034 | 0.343 ± 0.087 | 0.61 ± 0.2 | 0.58 ± 0.1 | 0.2 ± 0.019 | 0.212 ± 0.065 |
| *Mustelus mustelus* | 9.27 ± 1.35 | 11.02 ± 4.68 | - | - | - | - | - | - | - | - | - | - | - | - |
| *Mytilus galloprovincialis* | - | - | 0.079 ± 0.067 | 0.045 ± 0.0 | 0.435 ± 0.0 | 0.16 ± 0.0 | - | - | 0.3 ± 0.208 | 0.052 ± 0.0 | 0.32 ± 0.08 | 0.11 ± 0.06 | - | - |
| *Nassarius lima* | - | - | - | - | - | 0.112 ± 0.0 | - | - | - | - | - | - | - | - |
| *Nassarius mutabilis* | - | - | - | - | - | - | - | - | - | - | 0.145 ± 0.0 | 0.07 ± 0.0 | - | - |
| *Nassarius reticulatus* | - | 0.07 ± 0.0 | - | - | - | - | - | - | - | - | 0.609 ± 0.3 | 0.09 ± 0.05 | - | - |
| *Natica* spp | - | - | - | - | - | - | - | - | - | - | 0.145 ± 0.0 | - | - | - |
| *Nephrops norvegicus* | - | - | 0.035 ± 0.016 | 0.241 ± 0.0 | - | 0.153 ± 0.0 | - | 0.01 ± 0.0 | - | - | - | - | - | - |
| *Nucula sulcata* | - | - | - | 0.159 ± 0.0 | 0.141 ± 0.0 | - | - | - | - | 0.015 ± 0.0 | - | - | 0.1 ± 0.046 | 0.164 ± 0.069 |
| *NUDIBRANCHIA NDD* | 0.21 ± 0.04 | 0.26 ± 0.15 | 0.053 ± 0.0 | - | 0.124 ± 0.068 | 0.549 ± 0.0 | 0.51 ± 0.0 | 0.03 ± 0.0 | - | - | - | - | - | - |
| *Ocnus planci* | 2.61 ± 0.59 | 3.04 ± 0.56 | 0.079 ± 0.016 | 0.055 ± 0.008 | 0.448 ± 0.162 | 0.821 ± 0.395 | 0.26 ± 0.0 | 1.03 ± 0.47 | 0.2 ± 0.019 | 0.105 ± 0.081 | 0.18 ± 0.05 | 0.06 ± 0.0 | - | - |
| *Ophidion barbatum* | - | - | - | - | - | - | - | - | - | - | - | - | 0.5 ± 0.0 | - |
| *Ophiothrix* spp | 5.13 ± 1.22 | 7.63 ± 4.49 | 0.091 ± 0.0 | 0.455 ± 0.0 | 0.016 ± 0.0 | - | - | - | - | - | - | - | - | - |
| *Ophiura ophiura* | 0.34 ± 0.16 | 3.68 ± 3.23 | - | - | - | - | - | - | - | - | 0.036 ± 0.0001 | - | - | - |
| *Ophiura spp* | - | - | 0.002 ± 0.0 | 0.005 ± 0.0 | - | - | - | - | - | 0.017 ± 0.0 | - | - | - | - |
| *Ostrea edulis* | - | - | 0.467 ± 0.0 | 0.286 ± 0.194 | - | - | - | 1.25 ± 0.0 | - | - | 1.122 ± 0.0 | 0.72 ± 0.0 | - | - |
| *Paeneus spp* | - | - | - | - | - | - | - | - | - | - | - | - | 0.01 ± 0.0 | 0.014 ± 0.0 |
| *Pagellus acarne* | 11.12 ± 10.88 | 11.75 ± 11.63 | 0.063 ± 0.021 | 0.155 ± 0.051 | - | - | - | - | - | - | - | - | - | - |
| *Pagellus bogaraveo* | - | - | - | - | 0.819 ± 0.0 | - | - | - | - | - | - | - | - | - |
| *Pagellus erythrinus* | 10.51 ± 0.0 | 4.8 ± 3.68 | 0.452 ± 0.325 | 0.111 ± 0.023 | 1.346 ± 0.396 | 1.643 ± 0.419 | - | - | 0.11 ± 0.022 | 0.192 ± 0.048 | 0.06 ± 0.03 | - | 0.2 ± 0.077 | - |
| *Pagellus* spp | - | - | - | - | - | - | - | - | - | - | - | 0.06 ± 0.02 | - | - |
| *Paguristes oculatus* | 2.59 ± 0.65 | 1.06 ± 0.36 | 0.121 ± 0.025 | 0.258 ± 0.084 | - | - | - | - | - | - | - | - | - | - |
| *Pagurus excavatus* | - | - | - | - | - | 0.259 ± 0.112 | - | - | - | 0.061 ± 0.0 | 0.025 ± 0.0 | - | - | - |
| *Pagurus* spp | - | - | - | - | - | 0.082 ± 0.037 | 0.46 ± 0.0 | 0.27 ± 0.2 | - | - | - | - | - | - |
| *Paphia aurea* | - | - | 0.01 ± 0.0 | - | - | - | - | - | - | - | 0.014 ± 0.0 | - | - | - |
| *Parapenaeus longirostris* | - | - | 0.269 ± 0.047 | 0.38 ± 0.094 | 0.712 ± 0.175 | 0.975 ± 0.188 | - | 0.13 ± 0.0 | 0.17 ± 0.028 | 0.205 ± 0.045 | - | - | 1 ± 0.211 | 1.985 ± 0.345 |
| *Parthenope angulifrons* | 0.64 ± 0.23 | 1.31 ± 0.95 | 0.308 ± 0.183 | 0.524 ± 0.235 | - | - | - | - | - | - | - | - | - | - |
| *Pennatula rubra* | - | - | 0.009 ± 0.002 | 0.047 ± 0.014 | 0.03 ± 0.005 | 0.1 ± 0.038 | - | - | - | 0.025 ± 0.0 | - | - | - | - |
| *Phallusia mamillata* | 8.8 ± 3.12 | 2.46 ± 0.97 | 0.651 ± 0.222 | 1.087 ± 0.46 | 6.157 ± 2.621 | 8.529 ± 3.247 | - | 0.07 ± 0.0 | - | - | - | - | - | - |
| *Philine quadripartita* | - | - | - | - | - | - | - | - | - | - | 0.895 ± 0.3 | 0.16 ± 0.04 | - | - |
| *Phycis blennoides* | - | - | 0.189 ± 0.011 | 0.06 ± 0.0 | - | - | - | - | - | - | - | - | - | - |
| *Phycis phycis* | - | - | - | - | - | - | 0.32 ± 0.0 | - | - | - | - | - | - | - |
| *Phycis* spp | - | - | 0.124 ± 0.026 | 0.111 ± 0.058 | - | - | - | - | - | - | - | - | - | - |
| *Pilumnus spinifer* | 0.46 ± 0.13 | 0.73 ± 0.55 | - | - | - | - | - | - | - | 0.051 ± 0.0 | - | - | - | - |
| *PORIFERA NDD* | 19.13 ± 5.11 | 9.05 ± 2.26 | 0.319 ± 0.218 | 0.138 ± 0.043 | 0.407 ± 0.143 | 0.92 ± 0.263 | - | - | - | - | - | - | - | - |
| *Psammechinus microtuberculatus* | 27.47 ± 9.47 | 14.05 ± 9.15 | - | - | - | - | - | - | - | - | - | - | - | - |
| *Raja asterias* | 7.55 ± 0.0 | - | - | - | - | - | - | - | - | - | - | - | - | - |
| *Raja clavata* | - | - | - | - | - | - | 0.13 ± 0.0 | 0.1 ± 0.0 | - | - | - | - | - | - |
| *Raja miraletus* | - | - | - | - | - | 0.016 ± 0.0 | - | - | - | - | - | - | - | - |
| *Sardina pilchardus* | 2.19 ± 0.53 | - | 0.407 ± 0.116 | 0.698 ± 0.323 | 0.375 ± 0.17 | 0.149 ± 0.087 | 0.55 ± 0.01 | 2.99 ± 1.07 | 0.38 ± 0.092 | 0.414 ± 0.056 | 3.104 ± 0.7 | 4.02 ± 1 | 0.7 ± 0.327 | 0.822 ± 0.488 |
| *Scaphander lignarius* | - | - | - | 0.038 ± 0.0 | - | - | - | - | - | - | - | - | - | - |
| *Schizaster canaliferus* | - | - | - | - | - | - | - | - | - | - | - | 0.35 ± 0.0 | - | - |
| *Scomber scombrus* | - | - | - | - | - | - | - | - | - | 0.126 ± 0.0 | - | - | - | 1.095 ± 0.0 |
| *Scorpaena notata* | - | - | 0.186 ± 0.06 | 0.251 ± 0.073 | 0.33 ± 0.094 | 0.269 ± 0.054 | 0.44 ± 0.0 | - | 0.15 ± 0.043 | 0.342 ± 0.145 | - | - | 0.5 ± 0.0 | 0.206 ± 0.065 |
| *Scyliorhinus canicula* | - | - | 0.162 ± 0.0 | 0.17 ± 0.068 | 0.085 ± 0.01 | 1.529 ± 0.0 | - | - | - | - | - | - | - | - |
| *Sepia elegans* | - | - | 0.06 ± 0.017 | 0.093 ± 0.025 | 0.23 ± 0.05 | 0.385 ± 0.082 | 0.18 ± 0.0 | 0.15 ± 0.05 | - | - | - | - | 0.2 ± 0.06 | 0.142 ± 0.051 |
| *Sepia officinalis* | - | - | - | - | - | 0.593 ± 0.0 | - | - | 0.07 ± 0.047 | - | - | - | - | - |
| *Sepiola rondeleti* | 0.04 ± 0.0 | 0.09 ± 0.0 | 0.049 ± 0.009 | 0.048 ± 0.012 | - | - | 0.04 ± 0.0 | 0.03 ± 0.02 | - | - | - | - | 0.1 ± 0.042 | 0.132 ± 0.092 |
| *Serranus hepatus* | - | - | 0.386 ± 0.048 | 0.557 ± 0.084 | 0.91 ± 0.17 | 1.152 ± 0.315 | 0.08 ± 0.0 | 0.25 ± 0.08 | 0.24 ± 0.057 | 0.184 ± 0.047 | 0.859 ± 0.0 | - | 0.5 ± 0.132 | 0.555 ± 0.109 |
| *Sicyonia carinata* | 0.89 ± 0.85 | - | - | - | - | - | - | - | - | - | - | - | - | - |
| *Sipunculus* spp | - | - | - | - | - | 0.016 ± 0.0 | - | - | - | - | - | - | - | - |
| *Solea solea* | - | - | - | - | - | - | - | - | - | - | - | 1.26 ± 0.7 | 0.08 ± 0.032 | - |
| *Solecurtus strigilatur* | - | 1.61 ± 0.0 | - | 0.054 ± 0.0 | - | - | - | - | - | - | - | - | - | - |
| *Solenocera membranacea* | - | - | - | - | - | - | 0.12 ± 0.04 | 0.18 ± 0.07 | - | - | - | - | - | - |
| *Sparus aurata* | 0.8 ± 0.0 | 2.18 ± 0.0 | - | - | - | - | - | - | - | - | - | - | - | - |
| *Sphaerechinus granularis* | 2.61 ± 0.0 | - | - | - | - | - | - | - | - | - | - | - | - | - |
| *Sphyraena sphyraena* | - | - | - | - | - | - | - | - | - | - | - | 1.18 ± 0.0 | - | - |
| *Spicara maena* | 4.61 ± 2.08 | 5.09 ± 0.0 | - | 0.072 ± 0.0 | 0.52 ± 0.253 | 0.654 ± 0.244 | - | 1.13 ± 0.0 | 0.19 ± 0.0 | 0.104 ± 0.0 | - | 0.12 ± 0.0 | 0.3 ± 0.058 | 0.235 ± 0.035 |
| *Spicara smaris* | - | - | 0.057 ± 0.011 | 0.11 ± 0.056 | - | - | - | - | - | - | - | - | - | - |
| *Sprattus sprattus* | - | - | - | - | - | - | - | 0.76 ± 0.17 | 0.06 ± 0.0 | 0.136 ± 0.0 | - | - | - | - |
| *Squilla mantis* | 0.57 ± 0.01 | 0.68 ± 0.12 | 0.03 ± 0.011 | 0.021 ± 0.004 | 0.116 ± 0.032 | 0.545 ± 0.224 | 8.75 ± 2.8 | 2.1 ± 0.93 | 0.16 ± 0.088 | 0.348 ± 0.122 | 7.28 ± 1 | 5.19 ± 0.9 | 0.7 ± 0.595 | 0.364 ± 0.297 |
| *Sternapsis scutata* | - | - | - | - | - | - | - | - | - | 0.002 ± 0.0 | - | - | 0.03 ± 0.006 | - |
| *Stichopus regalis* | - | - | - | - | 1.399 ± 0.941 | - | - | - | - | - | - | - | - | - |
| *Suberites domuncula* | 12.28 ± 3.22 | 11.53 ± 4.19 | 0.074 ± 0.041 | 0.157 ± 0.088 | - | - | 0.02 ± 0.0 | 0.32 ± 0.12 | - | - | - | - | - | - |
| *Symphodus ocellatus* | - | - | 0.004 ± 0.0 | - | - | - | - | - | - | - | - | - | - | - |
| *Tellina* spp | - | - | 0.004 ± 0.0 | - | - | - | - | - | - | - | - | - | - | - |
| *Tethya aurantium* | 2.17 ± 0.57 | 1.99 ± 1.25 | - | - | - | - | - | - | - | - | - | - | - | - |
| *Torpedo marmorata* | - | - | - | - | 0.884 ± 0.0 | - | - | - | - | - | - | - | - | - |
| *Trachinus draco* | - | - | 0.055 ± 0.0 | - | - | 1.514 ± 0.0 | - | - | - | - | - | - | - | 0.248 ± 0.0 |
| *Trachurus mediterraneus* | 0.77 ± 0.0 | 1.46 ± 0.24 | 0.532 ± 0.0 | 2.016 ± 0.0 | - | - | 0.06 ± 0.0 | 0.48 ± 0.15 | - | - | 0.135 ± 0.0 | - | - | - |
| *Trachurus spp* | - | - | - | - | 0.379 ± 0.076 | 1.111 ± 0.367 | - | - | 0.15 ± 0.107 | 0.24 ± 0.0 | 0.347 ± 0.05 | 1.03 ± 0.5 | 3 ± 1.18 | 1.228 ± 0.323 |
| *Trachurus trachurus* | 15.22 ± 14.51 | 22.72 ± 0.0 | 0.518 ± 0.177 | 0.843 ± 0.179 | - | - | - | - | 0.05 ± 0.0 | - | - | - | - | - |
| *Trachythyone elongata* | - | - | - | - | - | - | - | 0.06 ± 0.0 | 0.04 ± 0.011 | 0.022 ± 0.0 | 0.017 ± 0.008 | - | 0.1 ± 0.054 | 0.06 ± 0.013 |
| *Trachythyone tergestina* | - | - | 0.021 ± 0.003 | 0.007 ± 0.0 | - | - | 0.06 ± 0.0 | 0.09 ± 0.04 | - | - | - | - | - | - |
| *Trisopterus minutus capelanus* | - | - | 0.255 ± 0.063 | 0.27 ± 0.066 | 1.627 ± 0.298 | 1.702 ± 0.26 | - | 0.22 ± 0.1 | 0.11 ± 0.016 | 0.094 ± 0.0 | - | - | 0.8 ± 0.179 | 0.32 ± 0.122 |
| *Trophonopsis muricata* | - | - | - | - | - | - | 0.03 ± 0.0 | - | - | - | - | - | - | - |
| *Turritella communis* | - | - | 0.028 ± 0.008 | 0.085 ± 0.024 | - | - | 0.27 ± 0.23 | 0.18 ± 0.05 | 0.12 ± 0.041 | 0.564 ± 0.237 | 0.03 ± 0.008 | 0.03 ± 0.008 | - | - |
| *Uranoscopus scaber* | - | - | 0.244 ± 0.0 | 0.204 ± 0.081 | 0.267 ± 0.0 | - | - | - | - | - | - | 0.25 ± 0.0 | - | - |
| *Virgularia mirabilis* | - | - | 0.003 ± 0.001 | 0.006 ± 0.002 | - | - | - | 0.05 ± 0.01 | - | - | - | - | - | - |
| *Zeus faber* | - | - | 0.056 ± 0.0 | 0.035 ± 0.0 | - | - | - | - | - | - | - | - | - | - |
