# Supplementary material for: Bottom trawl catch comparison in the Mediterranean Sea: Flexible Turtle Excluder Device (TED) vs traditional gear
Source: PLoS One. 2019 Dec 4;14(12):e0216023. doi: 10.1371/journal.pone.0216023 (PMC6892479; doi:10.1371/journal.pone.0216023)
Supplement: S3 Table — Marine litter codes: A = PLASTIC: 01 = bottle; 02 = sheet; 03 = bag; 05 = fishing line (monofilament); 07 = synthetic rope; 08 = fishing net; 09 = cable ties; 10 = strapping band; 11 = crates and containers; 12 = mussel farming ropes; 13 = other. C = METAL: 01 = cans (food); 02 = cans (beverage); 03 = fishing related; 07 = cables; 08 = other. D = RUBBER: 01 = boots; 02 = balloons; 04 = tyre; 05 = glove; 06 = other. E = GLASS/CERAMIC: 01 = jar; 02 = bottle; 04 = other. F = NATURAL PRODUCTS: 01 = wood (processed); 02 = rope; 05 = other. G = MISCELLANEOUS: 01 = clothing/rags; 03 = other. DEBRIS: shells = empty shells; echinoderms = piece of sea urchins or dead sea urchins; wood = natural wood (branches or tree trunk); organic = unidentified organic material. (DOCX) [file pone.0216023.s003.docx]

**S3 Table:** **List of the discard species caught during the trials with associated average CPUE_W_ and standard errors. Marine litter codes as follow: A = Plastic: 01 = bottle; 02 = sheet; 03 = bag; 05 = fishing line (monofilament); 07 = synthetic rope; 08 = fishing net; 09 = cable ties; 10 = strapping band; 11 = crates and containers; 12 = mussel farming ropes; 13 = other. C = METAL: 01 = cans (food); 02 = cans (beverage); 03 = fishing related; 07 = cables; 08 = other. D = RUBBER; 01 = boots; 02 = balloons; 04 = tyre; 05 = glove; 06 = other. E = GLASS/CERAMIC: 01 = jar; 02 = bottle; 04 = other. F = NATURAL PRODUCT: 01 = wood (processed); 02 = rope; 05 = other. G = MISCELLANEUS: 01 = clothing/rags; 03 = other. Debris shell = empty shells; debris echinoderms = piece of sea urchins or dead sea urchins; debris wood = natural wood (branches or tree trunk); debris organic = unidentified organic material.**

| Material | AST TED | AST CTRL | AUD TED | AUD CTRL | GLA TED | GLA CTRL | JOA TED | JOA CTRL | PAL TED | PAL CTRL | RIM TED | RIM CTRL | TAR TED | TAR CTRL |
| --- | --- | --- | --- | --- | --- | --- | --- | --- | --- | --- | --- | --- | --- | --- |
| Debris_shells | 10 ± 3.13 | 18.94 ± 10 | 1.159 ± 0.26 | 1.725 ± 0.27 | 0.954 ± 0.233 | 1.43 ± 0.36 | 2.66 ± 0.637 | 0.9 ± 0.11 | 5.067 ± 0.818 | 4.31 ± 0.8 | 1.63 ± 0.487 | 0.87 ± 0.23 | 3.78 ± 0.783 | 4.21 ± 0.8 |
| Debris_echinoderms | - | - | 0.064 ± 0.0 | - | - | - | - | - | - | - | - | - | - | - |
| Debris_wood | 2 ± 0.32 | 1.49 ± 0.3 | 3.656 ± 0.445 | 7.295 ± 0.813 | 0.894 ± 0.156 | 2.66 ± 0.97 | 2.29 ± 0.633 | 2.58 ± 1.31 | - | 4.24 ± 1 | 1.51 ± 0.233 | 3.93 ± 0.95 | 2.7 ± 0.429 | 7.04 ± 1.927 |
| Debris_organic | 20 ± 10.16 | 4.8 ± 2 | 0.171 ± 0.0 | 0.178 ± 0.05 | - | - | - | - | 3.739 ± 0.545 | 1.62 ± 0.4 | 0.59 ± 0.097 | 0.69 ± 0.16 | 3.59 ± 0.853 | 4.01 ± 0.336 |
| MARLITTER_A01 | 0.1 ± 0.02 | 0.04 ± 0.0 | 0.057 ± 0.014 | 0.174 ± 0.045 | 0.146 ± 0.069 | 0.41 ± 0.16 | 0.2 ± 0.06 | 0.06 ± 0.02 | 0.078 ± 0.038 | 0.05 ± 0.01 | 0.05 ± 0.011 | 0.05 ± 0.01 | - | 0.12 ± 0.051 |
| MARLITTER_A02 | - | - | 0.01 ± 0.0 | 0.011 ± 0.0 | - | - | - | 0.58 ± 0.0 | - | - | - | - | 0.04 ± 0.0 | 0.1 ± 0.055 |
| MARLITTER_A03 | 0.2 ± 0.19 | 0.16 ± 0.08 | 0.088 ± 0.018 | 0.258 ± 0.081 | 0.056 ± 0.015 | 0.36 ± 0.13 | 0.06 ± 0.02 | 0.16 ± 0.06 | 0.059 ± 0.03 | 0.1 ± 0.04 | 0.02 ± 0.0 | 0.19 ± 0.0 | 0.05 ± 0.0 | - |
| MARLITTER_A05 | - | - | - | - | - | 0.04 ± 0.02 | - | - | - | - | - | - | - | 0.04 ± 0.0 |
| MARLITTER_A07 | 0.04 ± 0.0 | - | 0.012 ± 0.005 | 0.037 ± 0.019 | 0.08 ± 0.0 | 0.52 ± 0.43 | 0.82 ± 0.672 | 0.12 ± 0.04 | 0.066 ± 0.023 | 0.2 ± 0.1 | - | - | - | 0.16 ± 0.03 |
| MARLITTER_A08 | - | - | 0.089 ± 0.047 | 0.171 ± 0.165 | - | - | - | 0.45 ± 0.44 | - | - | - | - | - | - |
| MARLITTER_A09 | - | 0.02 ± 0.0 | 0.039 ± 0.0 | - | - | - | - | - | - | - | - | - | - | - |
| MARLITTER_A10 | - | 0.33 ± 0.0 | - | 0.006 ± 0.002 | - | - | - | - | - | - | - | - | - | - |
| MARLITTER_A11 | 0.002 ± 0.0 | 0.04 ± 0.0 | 0.036 ± 0.01 | 0.091 ± 0.025 | 0.009 ± 0.002 | 0.4 ± 0.29 | 0.01 ± 0.006 | 0.09 ± 0.06 | 0.005 ± 0.0 | - | 0.02 ± 0.0 | - | 0.03 ± 0.0 | 0.01 ± 0.005 |
| MARLITTER_A12 | - | - | 0.115 ± 0.082 | 0.069 ± 0.033 | 0.063 ± 0.026 | 0.28 ± 0.1 | 0.05 ± 0.007 | 0.18 ± 0.09 | 0.043 ± 0.004 | 0.05 ± 0.005 | 0.03 ± 0.008 | 0.12 ± 0.06 | 0.05 ± 0.009 | 0.05 ± 0.028 |
| MARLITTER_A13 | 0.04 ± 0.0 | - | 0.089 ± 0.046 | 0.577 ± 0.399 | 0.05 ± 0.014 | 0.19 ± 0.04 | 0.22 ± 0.0 | - | 0.123 ± 0.056 | 0.07 ± 0.02 | 0.07 ± 0.023 | 0.16 ± 0.05 | 0.02 ± 0.006 | 0.09 ± 0.041 |
| MARLITTER_C01 | - | 0.09 ± 0.0 | - | - | 0.056 ± 0.0 | 0.28 ± 0.23 | - | - | - | 0.02 ± 0.0009 | - | 0.04 ± 0.0 | - | - |
| MARLITTER_C02 | 0.03 ± 0.0 | 0.07 ± 0.006 | 0.076 ± 0.017 | 0.111 ± 0.041 | 0.017 ± 0.003 | 0.05 ± 0.02 | 0.04 ± 0.004 | - | 0.015 ± 0.0 | 0.2 ± 0.1 | 0.01 ± 0.0 | - | - | 0.02 ± 0.0 |
| MARLITTER_C03 | - | - | 0.081 ± 0.0 | - | - | - | - | - | - | - | - | - | - | - |
| MARLITTER_C07 | - | - | - | - | 0.022 ± 0.0 | - | - | - | - | - | - | - | - | - |
| MARLITTER_C08 | - | - | - | - | - | 0.03 ± 0.0 | - | - | - | - | - | - | - | - |
| MARLITTER_D01 | - | - | 0.684 ± 0.0 | 0.621 ± 0.0 | - | 0.57 ± 0.0 | - | - | - | - | - | 0.38 ± 0.0 | - | 0.42 ± 0.0 |
| MARLITTER_D02 | - | - | - | - | - | 0.31 ± 0.0 | - | - | - | - | - | - | - | - |
| MARLITTER_D04 | - | - | - | - | - | - | - | 4.36 ± 0.0 | - | - | - | - | - | - |
| MARLITTER_D05 | - | 0.28 ± 0.0 | 0.143 ± 0.064 | 0.101 ± 0.033 | 0.04 ± 0.0 | - | - | 0.17 ± 0.0 | - | 0.02 ± 0.0 | - | - | - | - |
| MARLITTER_D06 | - | - | 0.036 ± 0.0 | 0.16 ± 0.047 | 0.065 ± 0.0 | 0.41 ± 0.18 | 0.3 ± 0.095 | - | - | 0.2 ± 0.1 | - | - | - | 1.29 ± 0.0 |
| MARLITTER_E01 | - | - | - | - | - | - | - | - | - | 0.11 ± 0.0 | - | - | - | - |
| MARLITTER_E02 | - | - | 0.092 ± 0.024 | 0.346 ± 0.128 | - | 0.2 ± 0.02 | 0.25 ± 0.0 | - | 0.092 ± 0.0 | - | - | - | 0.16 ± 0.145 | - |
| MARLITTER_E04 | - | - | - | - | - | 2.5 ± 0.0 | - | - | - | - | - | - | - | - |
| MARLITTER_F01 | - | - | 0.084 ± 0.0 | - | - | - | - | - | - | - | - | - | - | - |
| MARLITTER_F02 | 0.5 ± 0.0 | 0.44 ± 0.4 | - | - | - | 0.11 ± 0.0 | 0.01 ± 0.0 | - | - | - | - | 0.16 ± 0.0 | - | - |
| MARLITTER_F05 | - | - | - | 2.918 ± 0.0 | 0.044 ± 0.0 | 0.03 ± 0.0 | - | 0.11 ± 0.0 | - | - | - | - | - | - |
| MARLITTER_G01 | - | - | 0.262 ± 0.102 | 0.691 ± 0.213 | 0.071 ± 0.0 | 0.27 ± 0.12 | 0.1 ± 0.0 | - | - | - | - | - | - | 0.03 ± 0.014 |
| MARLITTER_G03 | - | - | 0.128 ± 0.072 | 0.624 ± 0.388 | - | 0.54 ± 0.0 | 0.09 ± 0.0 | - | - | 0.16 ± 0.0 | - | - | - | - |
