# Supplementary figures and images for: Bottom trawl catch comparison in the Mediterranean Sea: Flexible Turtle Excluder Device (TED) vs traditional gear
Source: PLoS One. 2019 Dec 4;14(12):e0216023. doi: 10.1371/journal.pone.0216023 (PMC6892479; doi:10.1371/journal.pone.0216023)

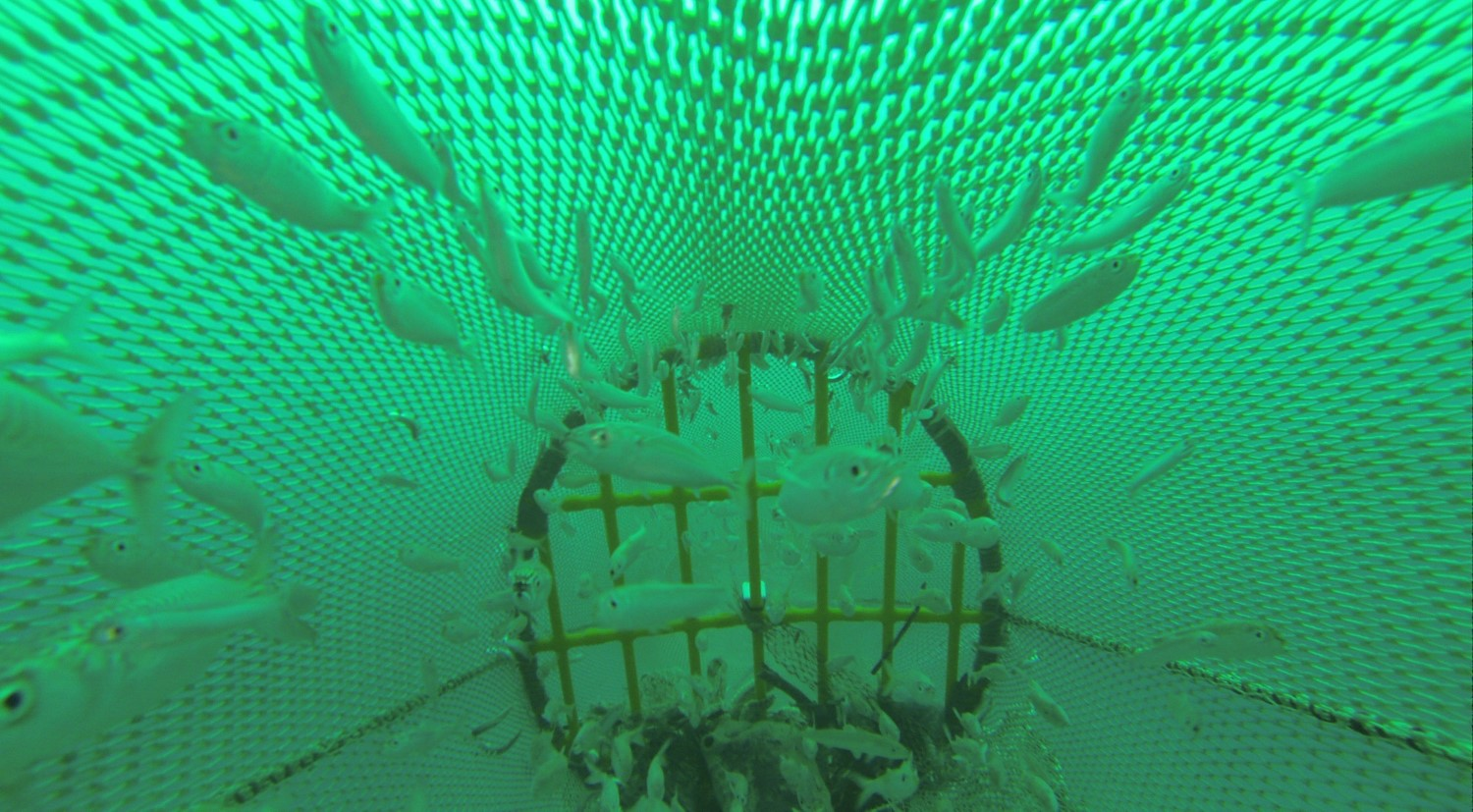

Supplement: S1 Fig — (TIF) [file pone.0216023.s004.tif]

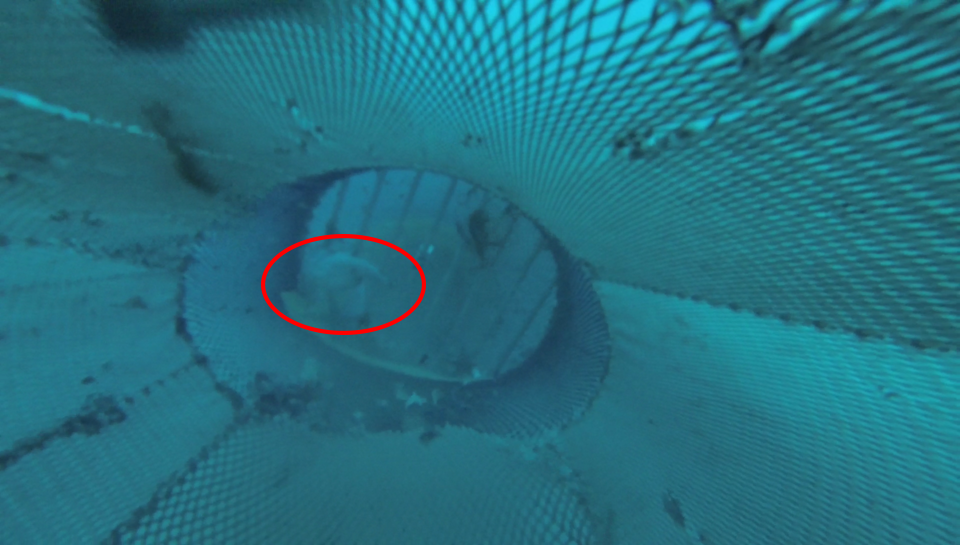

Supplement: S2 Fig — This is probably to be ascribed to the large head of this species. In some cases, angler fish were pushed into the grid by the hydrodynamic force, in other cases they rolled until they reached the opening on the upper side of the net, before the TED. Although the TED in the picture is not the same design as the one used in the trials, the flexible bars exert the same effect on this species. (TIF) [file pone.0216023.s005.tif]
